# Detecting qualitative changes in biological systems

**DOI:** 10.1038/s41598-020-62578-8

**Published:** 2020-05-18

**Authors:** Cristina Mitrea, Aliccia Bollig-Fischer, Călin Voichiţa, Michele Donato, Roberto Romero, Sorin Drăghici

**Affiliations:** 1 Wayne State University, Department of Computer Science, Detroit, 48202 USA; 2 Wayne State University, Department of Oncology, Detroit, 48201 USA; 3 Karmanos Cancer Institute, Detroit, 48201 USA; 4 Stanford University, Institute for Immunity, Transplantation and Infection, Stanford, 94305 USA; 5 Eunice Kennedy Shriver National Institute of Child Health and Human Development, NICHD/NIH/DHHS, Perinatology Research Branch, Division of Obstetrics and Maternal-Fetal Medicine, Division of Intramural Research, Detroit, 48201 USA; 6 University of Michigan, Department of Obstetrics and Gynecology, Ann Arbor, 48109 USA; 7 Michigan State University, Department of Epidemiology and Biostatistics, East Lansing, 48824 USA; 8 Wayne State University, Center for Molecular Medicine and Genetics, Detroit, 48201 USA; 9 Wayne State University, Department of Obstetrics and Gynecology, Detroit, 48201 USA

**Keywords:** Computational models, Data mining

## Abstract

Currently, most diseases are diagnosed only after significant disease-associated transformations have taken place. Here, we propose an approach able to identify when systemic qualitative changes in biological systems happen, thus opening the possibility for therapeutic interventions before the occurrence of symptoms. The proposed method exploits knowledge from biological networks and longitudinal data using a system impact analysis. The method is validated on eight biological phenomena, three synthetic datasets and five real datasets, for seven organisms. Most importantly, the method accurately detected the transition from the control stage (benign) to the early stage of hepatocellular carcinoma on an eight-stage disease dataset.

## Introduction

In most, if not all, non-trauma health-care cases, pathological conditions are defined by phenotypic or clinical changes. For example, cancer is usually diagnosed after the patient experiences symptoms caused by significant transformations in their physiology. However, the progression from a healthy state to one of disease is gradual, happening over a period of time. This is particularly true in the case of conditions such as cancer or neurodegenerative disorders, for which the onset of the underlying pathology is believed to begin much earlier than the clinical, detectable onset^1,2^. What if one could identify a departure from the healthy state well before a tumor is present, when changes can perhaps still be reversed? What if one could identify qualitative changes in the states of a biological system without even knowing what the states are? Here, we propose a technique that aims at identifying such qualitative changes without any a priori knowledge about the nature of the changes. The preliminary results herein demonstrate the potential of this approach using several datasets derived from eight biological phenomena and seven organisms.

The goal is to develop an approach that can detect qualitative changes in the system, where a qualitative change is defined as a change that involves observable macroscopic phenotypical or clinical changes. We should emphasize that no known approach is available to tackle this type of problems. There are no clearly defined states or classes available a priori, so no supervised machine learning approaches can be used. We would like to be able to detect changes as they happen if possible, without massive amounts of partially redundant data collected beforehand, so no unsupervised methods could be used to extract common features and build clusters. Here we are looking at a system without having a reference set of genes, so no enrichment approach will be useful. Finally, there is no predefined phenotype, and therefore no gene set analysis methods can be employed either. What we would like to achieve here is a method capable of (1) monitoring the activity of a system by taking periodic measurements and (2) detecting when a specific system undergoes a qualitative change without prior knowledge about it. To the best of our knowledge, no existing method could approach this task with a reasonable chance of success.

In this paper, we propose a qualitative change detection (QCD) approach, an analysis method that uses sequential measurements as described by a time series (or by progressive disease stages), together with all known interactions described by biological networks, and that applies an impact analysis approach to identify the time interval in which the system transitions to a different qualitative state.

In practical terms, the data to be analyzed is a time series of gene expression or any other sequential measurements of systemic states such as the one described in disease progression. Time-series data have been used in many ways, e.g. to infer information regarding regulatory mechanisms, the rate of change for a gene, the order in which genes are (de)activated, and the causal effects of gene expression changes^3^. Often, time series-data are used to extract gene profiles that can be be used to better understand the phenomena or phenotypes^4–7^. The analysis of time-series data can also be used to identify disease biomarkers either as a single gene, a group of genes, or a network of genes^8^.

**Figure 1:**
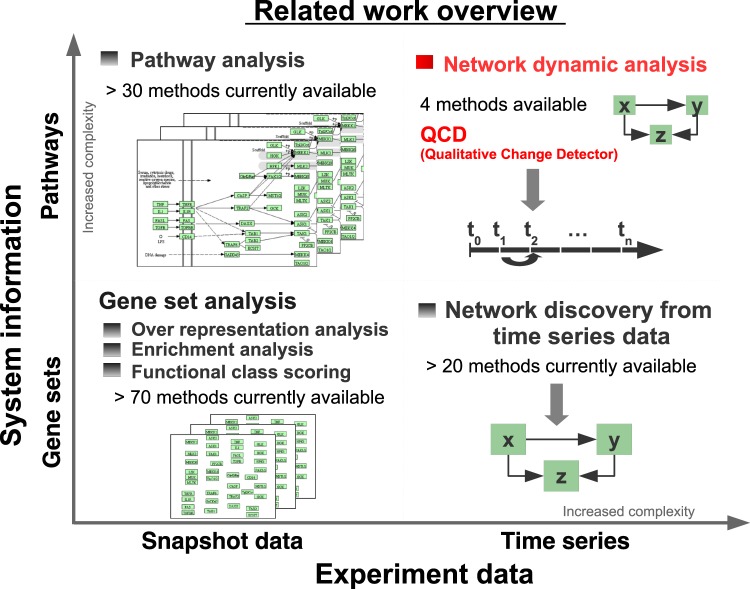
Overview of existing approaches as categorized by looking at the time component (horizontal axis) and the system information (vertical axis). From the time component perspective one can distinguish between two categories: snapshot data and time course data. Time course data is richer in information but also has increased complexity as opposed to snapshot data. From the system information perspective one could consider sets of genes together with their interactions (pathways) or without such interactions (gene sets). Pathways are much richer in information but also have increased complexity as opposed to gene sets. Based on these categories, the existing methods can be divided into the four groups shown, of which the gene set analysis is the most common, including more than 70 methods^64,65^. Gene set analysis takes as input a collection of gene sets and a snapshot of expression data that compares two phenotypes and ranks the gene sets based on their relevance to the phenotype computed by the analysis. Pathway analysis has the same workflow as the gene set analysis but also takes into consideration the interactions between the genes as described by the topology of the pathways^66,67^. Network discovery from time course data takes as input data collected at multiple time points and a set of genes and infers relations between the genes in the input set^68^. Network dynamic analysis is the most recent, and has only 4 existing methods^10–12,69^. Methods in this category (including the proposed method) use time series data and pathways to gain knew knowledge about the underlying phenomena.

In the landscape of analysis methods for high-throughput data (see Fig. 1), the proposed method falls under the category of dynamic network analysis. Other methods in the same category aim to either identify significantly perturbed systems^9^, time intervals with the highest difference in expression for each gene from a predefined set^10^, dynamic network biomarkers using local network entropy^11^, or time periods of differential gene expression using Gaussian processes^12^. However, all of these approaches perform comparisons between disease profiles and a reference profile (e.g. healthy). In the paradigm proposed here, none of these existing methods can be applied because the goal is to identify a transition to a qualitatively different state without knowing the gene expression profile of the new state, and hence, without the ability to make a comparison between the control and disease phenotypes.

A biological system is characterized by a tendency to reach and maintain a state of homeostatic balance, considered to be a stable state. An alteration made by internal or external stimuli can trigger the system to transition from one stable state to another, referred to as a qualitative change. Notably, any of the system components taken in isolation may not vary dramatically; however, the system as a whole may undergo a qualitative change. Conversely, in a resilient system, important variations of one or a group of components may happen without necessarily involving a qualitative systemic change. Importantly, most systems have built-in tolerance mechanisms such that the response to a stimulus is delayed until the signal is perceived as real in order to filter noise and to conserve the energy necessary to undergo a systemic change.

**Figure 2:**
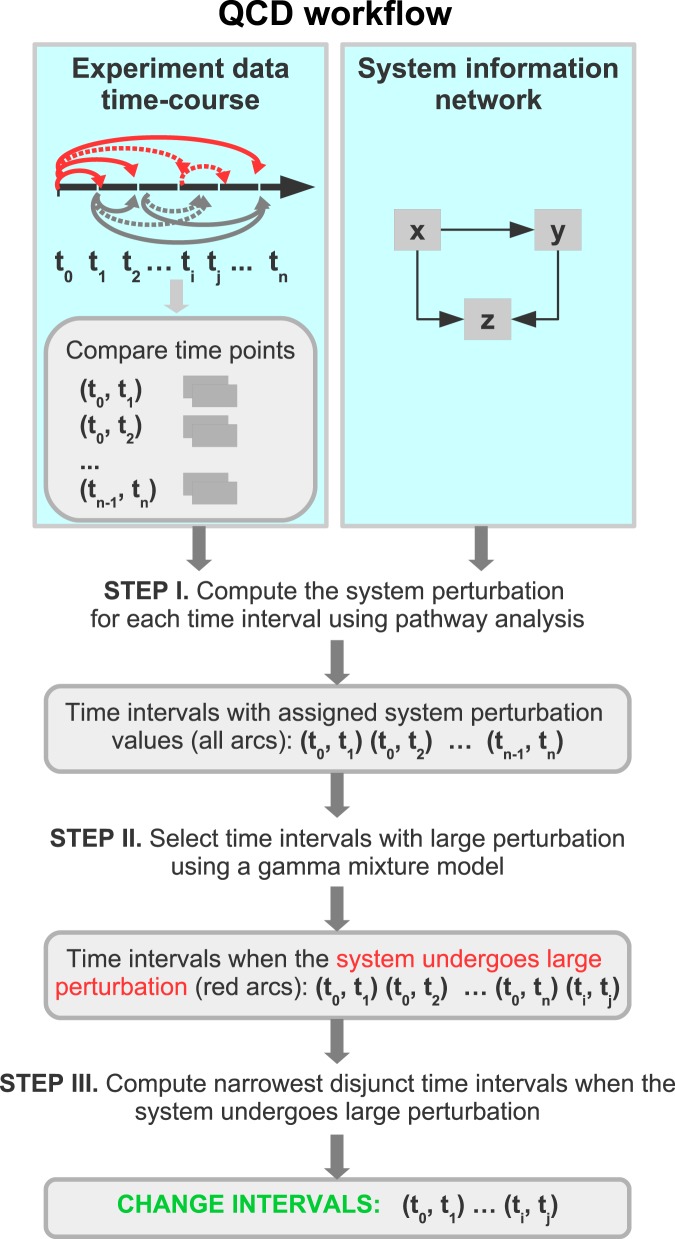
Workflow of the QCD method. The algorithm takes as input time series data and network(s) that models the biological system. The time series data is used to compare every pair of time points (time interval). In STEP I, a pathway impact analysis is used to compute a perturbation score for each comparison. In STEP II, an expectation maximization algorithm is employed to identify the parameters of a gamma mixture model and select the interval(s) when the system/pathway/network experienced a large perturbation. In STEP III, change intervals are selected by identifying the overlap of the set of intervals with large system perturbation and selecting the narrowest disjunct time intervals.

We developed and implemented a data analysis method capable of detecting qualitative changes in biological systems despite these challenges. The workflow of the analysis is summarized in Fig. 2. The input to QCD consists of: (i) time-series data and (ii) a network model of the biological system under study. QCD uses the input data to evaluate the system perturbation between each pair of time points/system states using a pathway impact analysis approach^13–16^. An expectation maximization algorithm is then used to separate large and small system perturbations values, thus identifying important differences between those states. Lastly, the analysis finds the disjunct overlaps of the intervals with large system perturbation that identify one or more time intervals during which the biological systems has undergone qualitative changes, referred to henceforth as change intervals.

## Results

Conventional approaches to the analysis of time series gene expression data are extremely useful tools to identify genes that are behaving in a similar way. However, these methods are not designed to identify systemic changes. The goal of the proposed approach is to identify transitions from one state to another, rather than study a particular state or a particular time profile. Our goal is to show that the proposed approach is able to identify such meaningful transitions across different organisms and various phenomena.

The analysis of eight well-studied phenomena was performed with the proposed method (QCD) for seven model organisms using both synthetic and real data. To assess the ability of QCD to detect qualitative changes, results were compared to prior knowledge of the phenomenon under study. QCD uses system knowledge, as described by a known gene signaling network or a map of neurons and their synaptic connections, as well as sequential measurements of the system components (genes or neurons). Data were obtained by measuring either the mRNA level of the genes involved in the system, in the case of real data, or generated based on equations describing the model of each organism, in the case of synthetic data.

The results of the analyses show that QCD can reliably identify the time interval during which a biological system goes from one qualitative state to another in response to organism development or to a shift in environmental conditions. We evaluate the method using phenomena that involve major physiological changes. We also evaluate the method for phenomena involving more subtle, yet important changes. Major physiological changes analyzed using synthetic data are *E. coli* flagellum building^17,18^ and *B. subtilis* sporulation^17,19^. The subtle change analyzed using synthetic data is *C. elegans* avoidance reflex^17,20^. Major physiological changes analyzed using real gene expression data are yeast sporulation^21^ and fruit fly pupariation^22^. More subtle changes analyzed using real gene expression data involve fruit fly ethanol exposure^23^.

QCD was compared with an existing method developed by Liu and colleagues used to detect network biomarkers and the pre-disease state (herein abbreviated DNBM)^11^. In addition to the six datasets mentioned above, we also ran QCD on the two datasets from the Liu *et al*. study. The first dataset is derived from a mouse study of exposure to a toxic gas (carbonyl chloride). Using these data QCD identified one qualitative change, before the exposure became lethal, preceding the pre-disease state detected by Liu *et al*. The second dataset contains data describing the progression of human hepatocellular carcinoma. Using these data, QCD identified a qualitative change from a benign stage (control) to a pre-malignant stage (high-grade dysplastic nodules), also preceding the pre-disease state detected by the Liu *et al*. study.

### Bacterium flagellum building

When in an environment lacking nutrients, the *E. coli* bacterium initiates the process of building a flagellum that will provide the motility necessary for finding an environment rich in nutrients.

**Figure 3:**
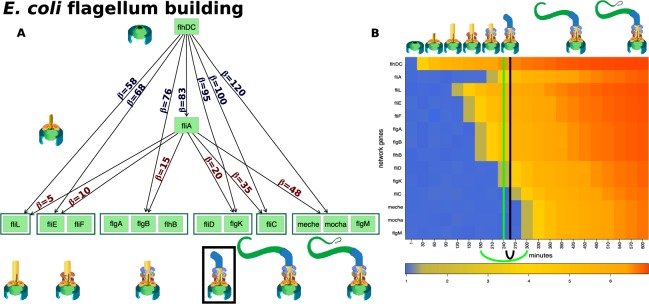
The input and results of the qualitative change detector (QCD) for the *E. coli* flagella building phenomenon. Panel (A) The multi-output coherent type 1 feed-forward loop (C1-FFL) network that describes the flagellum building, together with the activation thresholds ($\beta$ on the edge) for each of the six groups of genes (dark green boxes)^17,18^. The flagellum building is depicted in the cartoons matching the activation of each group of genes. The black box denotes building the flagellum hook which is the point of no return in this process and hence the real change interval that we aim to discover. Panel (B) The heatmap of the sampled data (input to QCD), and the real change interval (black arc below the heatmap and black vertical line positioned in the center of the interval) as described by literature. The change interval detected by QCD is shown by the green arc below the heatmap and the green vertical line positioned in the center of the interval (very close to the black line showing the actual point of no return). The stages of the flagella building are presented as cartoons in chronological order on the top part of the figure.

We analyzed the process of building the *E. coli* flagellar motor, using synthetic data and the flagellum building network^18^ (see Fig. 3A). Previous studies describe this network as a multi-output coherent type 1 feed-forward loop (C1-FFL)^18,24^. A C1-FFL is a network in which one gene activates another and, together, they activate another gene or (groups of) genes in the multi-output networks^24,25^.

The flagella building network is a generalization of the C1-FFL. In essence, the flagella building network is a multi-output C1-FFL in which the exact timing of the sequence of steps is controlled by the different activation thresholds (see the edge labels in Fig. 3A). These thresholds ensure that all the elements of the flagellum are built in a specific order so that it can properly assemble (e.g. the base of the structure must be in place before all other elements). Due to the different activation thresholds, a reverse order of the activation thresholds for $flhDC$ and $fliA$ yields a first-in first-out (FIFO) order in the gene transcription. This is typical of sensory transcription networks as a mechanism used to filter out (not react to) noise containing false positive signals of short duration.

Gene expression data was generated for the flagellum building network for a period of $10$ hours using a continuous function that models the protein accumulation and parameters from previous studies^17,18^. Samples were taken every 30 minutes leading to a gene expression time course dataset with 21 time points. Panel B in Fig. 3 shows the evolution of gene expression over time for the genes involved in this phenomenon.

Importantly, the organism commits to building the flagellum when the first hook of the flagellar motor starts to be built ($fliA$ reaches the threshold to regulate the next group of genes, $fliD$ and $flgK$)^18^. This is an important check point in the flagella building process as the assembly of the following component can still be halted if necessary^26^. However, after this checkpoint, the bacterium commits to building the flagellum (see the top of panel B in Fig. 3). For these reasons, the interval between 240 and 270 minutes can be considered the boundary that separates the two qualitatively different states: with and without flagellum. The goal of our approach is to find this interval without any knowledge about the phenomenon and with knowledge only from the gene expression data and the network of the system.

The *E. coli* flagellum construction is controlled by two transcription factors, $flhDC$ and $fliA$ (see Fig. 3A). The master regulator $flhDC$ activates $fliA$ and there is an $OR$ relationship through which these two master regulators activate the other genes in the network ($12$ genes). The genes are part of $6$ groups: (i) $fliL$, (ii) $fliE$ and $fliF$, (iii) $flgA$, $flgB$, $flhB$, (iv) $fliD$, $flgK$, (v) $fliC$, (vi) $meche$, $mocha$ and $flgM$.

**Figure 4:**
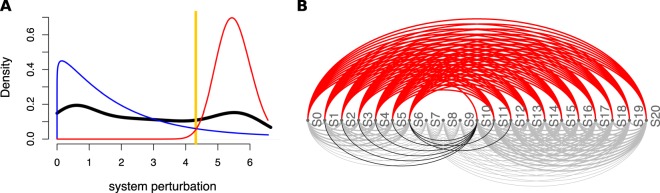
Identifying the qualitative change interval for the *E. coli* flagella building phenomenon. Panel (A) Identifying state comparisons involving large perturbations. The black line shows the observed density of the perturbation values for all pairwise comparisons of system states. We assume that some comparisons will be characterized by large perturbations, while others by small perturbations. A mixture of two gamma distributions are fitted to the observed data to yield the distributions of large (red) and small (blue) perturbation whose mixture best fits the observed data (red and blue lines). The intersection point (yellow vertical line) is the optimal threshold used to distinguish between the large and small perturbations. Panel (B) The arcplot of all comparisons performed by QCD between all pairs of system states. Red arcs, above the x axis, represent comparisons that show a large perturbation, while gray arcs, below the x axis, represent comparisons with a small perturbation. All the comparisons between states in the intervals S0–S6 and S10–S20 are associated with small perturbations. At the same time, the vast majority of all possible comparisons between any state in the interval S0–S6 and any state in the interval S10–S20 are associated with large perturbations. The black arcs are comparisons between a state in the interval S0–S6 and a state in the interval S10–S20 that are associated with small system perturbations. The smallest interval of overlapping large perturbation intervals, the interval between S6 and S10, is the detected change interval.

QCD compares all system states (time points) to each other using a pathway impact analysis. In essence, the state of the system at each time point is compared to the state at all other time points using a pathway impact analysis^13^ that takes into consideration all gene expression changes, the position of each gene on the pathway (Fig. 3A), and the type and direction of every interaction to determine if the state of the system was altered. The result of this impact analysis is a set of system perturbation factors that quantify the system perturbation. To determine the significant system perturbations, we assume there are two types of intervals: i) those with large perturbations between the states involved, and ii) those with small perturbations caused only by random fluctuations. We then use an expectation maximization algorithm to fit a gamma mixture model of two distributions to the perturbation factors (see Fig. 4). The intersection of the two distributions will be the optimal threshold that can be used to separate the large perturbations from the small perturbations as presented in Fig. 4A. Using this approach, we assign a “large” or “small” perturbation status to each comparison. Panel B in Fig. 4 shows all the state comparisons considered, in which the gray and black arcs show small perturbations and the red arcs show large perturbations between the states of the system at those time points.

In essence, most of the comparisons between any time point earlier than 180 mins and any time point after 300 mins show large perturbations (exceptions are marked by the black arcs). This suggests that a qualitative change of the system occurs between 180 and 300 mins, which is indeed the case. The real change takes place between 240 minutes, when $fliD$ and $flgK$ expression begins, and 270 minutes, when $fliA$ starts to regulate the next group of genes and the building of the first hook of the flagellar motor begins.

The identification of a change interval should be followed by an analysis of the states of the system before and after a change interval in order to gain insight into the system transition. Without loss of generality, we will consider the situation in which there is a single change interval, as in this dataset. Furthermore, we also assume that the system is in a stable state before and after the change interval. Under these circumstances, we can group the states in which the system is stable into meta-states.

A meta-state is a group of consecutive states where all comparisons between states within a meta-state have a small perturbation and all comparisons between states from a meta-state to states outside it (excluding the states in any change intervals) have a large perturbation.

The results shown in panel B of Fig. 4 suggest that states S0–S6 might form a meta-state, MS1. Similarly, the states S10–S20 might define a second meta-state, MS2. To investigate these potential meta-states, all comparisons (arcs) were studied from the perspective of the above definition of a meta-state. From this perspective, all these comparisons can be either consistent or inconsistent with the expectations noted above. This is a binary choice, and under the null hypothesis in which there are no meta-states, the probability that a comparison is consistent or not should be 0.5. Based on this framework, a binomial model can be used to calculate a p-value characterizing the amount of evidence that indicates the existence of a true meta-state (comparisons consistent with the definition vs. inconsistent comparisons). More details can be found in the Methods section, subsection “Meta-states statistical validation”. Groups of states with significant p-values will be reported as meta-states.

In this case, both groups of states identified by QCD had highly significant p-values: $p=5.44\times 1{0}^{-19}$ for meta-state 1 (S0–S6) and $p=3.61\times 1{0}^{-28}$ for meta-state 2 (S10–S20).

### Bacterium sporulation

When deprived of food, the *B. subtilis* bacterium turns into a spore, a robust structure able to survive in an environment lacking nutrients. This is a crucial feature that ensures the bacterium’s survival in an environment scarce in food in which it cannot survive in its active form.

**Figure 5:**
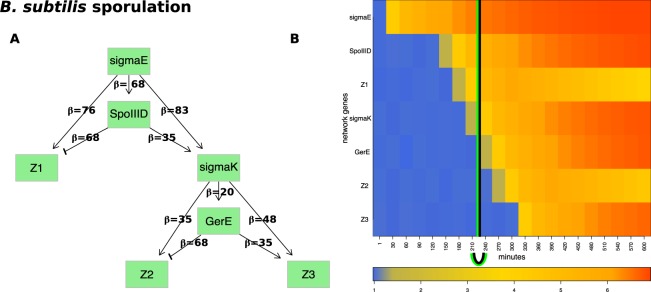
The input and results for QCD for *B. subtilis* sporulation. Panel (A) shows the sporulation network. The genetic network represented by the two coherent type 1 feed-forward loops (C1-FFLs) and two incoherent type-1 FFLs (I1-FFLs) that describe the sporulation network as reported in previous studies^17,19^. Panel (B) shows the heatmap of the sampled data. The real change interval is shown by the black arc below the heatmap (black vertical line positioned at the average of the interval limits) as described by literature. The change interval detected by the proposed method is shown by the green arc (green vertical lines positioned at the average of the interval limits), match perfectly with the actual timing of these events.

Compared to the *E. coli* flagellum-building network, which includes only activation signals, the network controlling sporulation also includes repression signals (Fig. 5A). This network has a hierarchical structure that consists of four transcription factors: $sigmaE$, $sigmaK$, $GerE$, $SpoIIID$ and three groups of genes $Z1$, $Z2$, $Z3$. This network is comprised of two network motifs, each of them represented by two networks. The two coherent feed-forward loops (C1-FFLs) aim at $sigmaK$ and $Z3$, respectively, while the two incoherent type-1 feed-forward loops (I1-FFLs) are centered around $Z1$ and $Z2$, respectively. The C1-FFLs are denoted as coherent because their central genes, $sigmaK$ and $Z3$, respectively, receive activation signals from both genes upstream of each. Specifically, $sigmaK$ receives activation signals from both $SpoIIID$ and $sigmaE$, while $Z3$ receives activation signals from both $GerE$ and $sigmaK$. In contrast, the incoherent network is characterized by a gene that receives one activation and one repression signal from the two genes immediately upstream of the target gene. For example, $Z1$ is activated by $sigmaE$ but repressed by $SpoIIID$.

The gene expression data was sampled from the spore formation network for a period of $10$ hours using a continuous function that models the protein accumulation and parameters observed in previous studies^17,19^. Samples were taken every 30 minutes leading to a gene expression time course dataset with 21 time points.

Importantly, the organism commits to the spore formation when the second suppressor ($GerE$) is expressed (4 h = 240 min)^19^. In turn, $GerE$ is regulated by $sigmaK$ which also regulates the communication between the mother cell and the spore through a checkpoint that is crucial for the formation of viable spores. Hence, the true interval of change is the interval between 210 minutes, when $sigmaK$ shows the first change in expression, and 240 minutes, when $GerE$ shows the first change in expression.

Our method was applied on the sporulation network and the synthetic gene expression dataset obtained by the above sampling. In these data, QCD identified one change interval (210–240 min) (Fig. 5B). The detected interval exactly matches the time interval between the time when the spore formation starts ($GerE$ is being expressed) and up to the moment when the next group of sporulation genes ($Z2$) is activated.

We also evaluated the two groups of system states: before the change interval (0–210 min) and after the change interval (240–600 min), as potential meta-states MS1 and MS2, respectively. The p-value for each was highly significant: $p=2.31\times 1{0}^{-19}$, for MS1, and $p=6.23\times 1{0}^{-32}$, for MS2. These p-values validate the hypothesis that these are true meta-states. Interestingly, these meta-states can be mapped to the rod-shaped bacterium form and the endospore form, respectively, while the detected change interval can be associated with the process of spore formation. These results are consistent with previous studies and interpretations^19^. Specifically, the *sigmaK* factor expression was identified as the critical control element in the regulatory mechanisms and the coordination of spore formation between the mother cell and forespore. In particular, *sigmaK* activates *GerE* which in turn triggers the expression of the last set on genes. For this reason, the true time point that can be considered as separating the rod-shaped bacterium from the endospore state is the point in which *sigmaK* becomes expressed (shown by the black line in Fig. 5). Before the change interval, the bacterium preserved most of its initial characteristics, while after this interval, the bacterium assumed most of the characteristics of an endospore. During the change interval, the system exhibited characteristics of both “spore” and “no spore” states. To conclude, in the study of Bacterium sporulation phenomenon, DQC accurately identified the transition from the rod-shaped bacterium form (no spore) to the endospore (spore) form.

### Worm avoidance reflex

**Figure 6:**
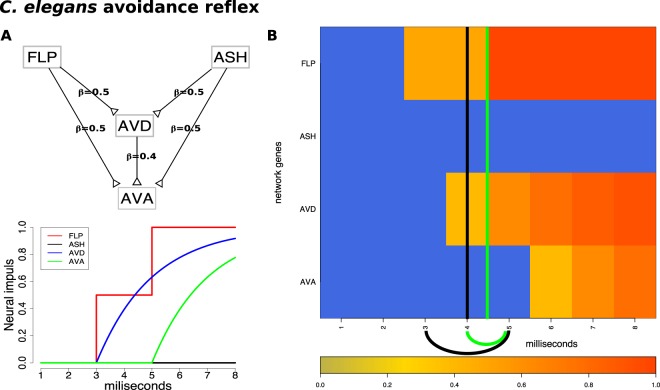
The input and results for QCD for the *C. elegans* avoidance reflex. Panel (A) top: The network that describes the avoidance reflex network as presented in previous studies^17,20^ is a multi-input coherent type 1 feed-forward loop (C1-FFL) with two inputs. Synaptic weights are marked by the $\beta$ values on the edges. Panel (A) bottom: The signal dynamics of the avoidance reflex network. Panel (B) The heatmap of the sampled data (which is the input to QCD) and the real change interval shown here by the black arc below the heatmap (black vertical line positioned in the center of the interval) as described by literature. The change interval detected by the proposed method and shown by a green arc below the heatmap (vertical lines positioned in the center of the interval), matches almost perfectly with the actual timing of these events.

A phenomenon involving more subtle changes is the nociception reflex. Nociception is a sensory process that allows the detection of harmful stimuli and activates a reflex response to move a part of the body or the whole body away from the stimulus. Nociceptors are present in fish, worms, and fruit flies, among others, and help trigger an avoidance reflex such as a backward movement. In the roundworm (*C. elegans*), the avoidance reflex network is composed of two parallel receptor neurons that communicate with two sequential command neurons (Fig. 6A).

The *C. elegans* avoidance reflex network is a generalization of the C1-FFL in the form of a multi-input C1-FFL. As previously described, C1-FFL is a network of three nodes in which one node activates another and, together, they activate another node^24,25^. In multi-input C1-FFL networks, the initial activation is performed by multiple nodes or groups of nodes rather than by just one node. $ASH$ is the main nociceptor and triggers avoidance behavior in response to harmful stimuli such as the nose touch and volatile chemicals. $FLP$ is a sensory neuron triggered by painful, heat-related stimuli or mechanical stimuli, such as a harsh nose touch, that initiates the nematode’s backward movement. $AVD$ is a command interneuron that functions as a modulator for backward locomotion induced by a head touch. Neurons $AVA$ and $AVD$ drive the worm’s backward movement.

Neuronal signal data was generated for the avoidance reflex network over a period of $8$ milliseconds, using a continuous function that models the signal processing and parameters observed in previous studies^17,20^. Samples were taken every millisecond leading to a time course dataset with eight time points.

The nematode commits to the backward movement at 3ms, which is the moment the nose touch ($FLP$ - spiking function) reaches the threshold to trigger the second command interneuron ($AVD$). The movement starts at 5ms when the $AVA$ neuron starts firing^20^. The two time-points mark the 3 to 5 ms time interval which is the real change interval. Using these data, QCD identified the narrower 4 ms to 5 ms interval (Fig. 6B).

In addition, the two groups of system states, before and after the change interval, were evaluated as potential meta-states. The p-values for the two groups of states are highly significant: $p=4.28\times 1{0}^{-4}$ for meta-state 1 and $p=4.28\times 1{0}^{-4}$ for meta-state 2. In summary, in the case of the avoidance reflex, the detected change interval is a transition between “no movement” and “moving backward” meta-states.

**Figure 7:**
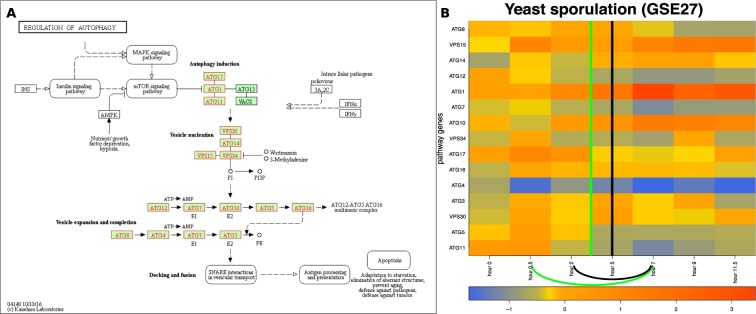
The input and results for QCD for yeast sporulation. The input is the regulation of autophagy pathway from KEGG^29– 31^ (sce04140)*, in Panel (A), and gene expression data from the GEO dataset GSE27, in Panel (B). The data captures the sporulation phenomenon, specifically the transition from diploid cells through meiosis to the spore cells. Panel (B) shows the heatmap of the time course (0 to 11.5 hours) for the measured KEGG pathway genes (in red), with the change interval detected for the phenomenon (green arc and the green vertical line in the center of the interval (0.5–7 h)), as well as the real change interval (black arc and the black vertical line in the center of the interval (2–7 h)). *For details about the pathway notations see the KEGG legend at: https://www.genome.jp/kegg/document/help_pathway.html.

**Figure 8:**
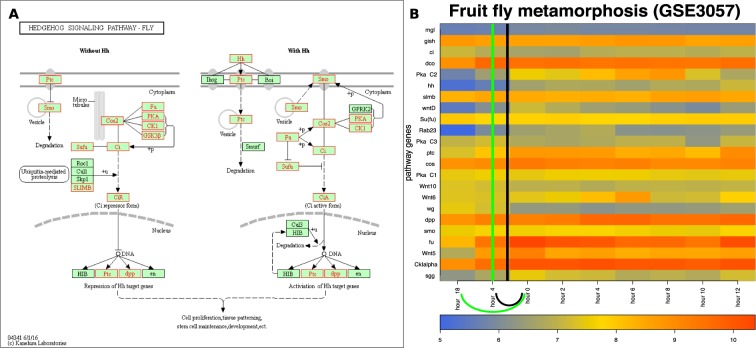
The input and results for QCD on fruit fly metamorphosis (pupariation). The input is the Hedgehog pathway from KEGG^29– 31^ (dme04340), in Panel (A), and gene expression data from the GEO dataset GSE3057, in Panel (B). The data captures the pupariation phenomenon, specifically transition from the end of the larva stage through the prepupa stage and to the beginning of the pupa stage of the fruit fly. Panel (B) shows the heatmap of the time course ($-18$ to 12 hours) for the measured KEGG pathway genes (in red), with the change interval detected for the phenomenon (green arc and the green vertical line in the center of the interval ($-18$–0 h)), as well as the real change interval (black arc and the black vertical line in the center of the interval ($-4$–0 h)).

**Figure 9:**
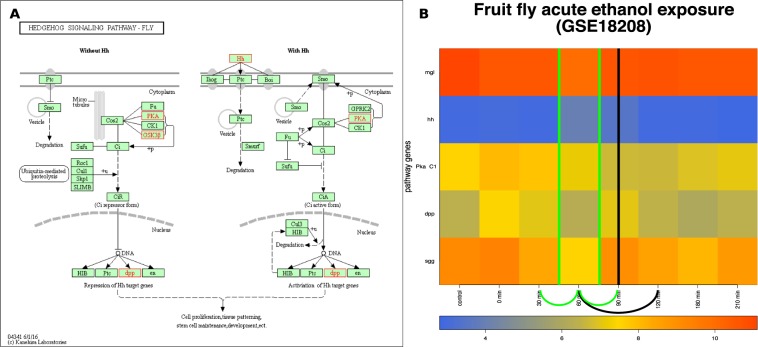
The input and results for QCD on fruit fly ethanol exposure. The input is the Hedgehog pathway from KEGG^29– 31^ (dme04340) in Panel (A), and gene expression data from GEO GSE18208, in Panel (B). The data captures the acute ethanol exposure phenomenon, specifically transition from the “sober” stage through the “drunk” stage and back to the “sober” stage. Panel (B) shows the heatmap of the time course (control, 0 to 3.5 hours) for the measured KEGG pathway genes (in red), with the change interval detected for the phenomenon (green arc and the green line in the center of the intervals (0.5–1 h) and (1–1.5 h), as well as the real change interval (black arc with a black line in the center of the interval (1–2 h)).

Results of the first three case studies, for which we used synthetic data, proved that QCD can be quite accurate. However, in practice, the data from real biological experiments can be very noisy. In order to investigate the capabilities of this approach to detect the correct change interval from real gene expression data, we used datasets collected from three different experiments: yeast sporulation, fruit fly metamorphosis, and acute ethanol exposure (see Figs. 7, 8 and 9). All data are available in the public domain in the Gene Expression Omnibus (GEO)^27,28^. Again, we chose different phenomena and different model organisms for a thorough method evaluation.

### Baker’s yeast sporulation

Starvation for nitrogen and carbon sources (high stress) induces meiosis and spore formation in diploid yeast (*S. cerevisiae*) cells. Stress-tolerant haploid spores are formed through cell division (meiosis) within the mother cell. This is a qualitative and obvious physiological change in yeast cells adapting to their environment. The sporulation process has been thoroughly studied and is well understood^21^, which makes it a good candidate on which to validate QCD.

We used the Kyoto Encyclopedia of Genes and Genomes (KEGG)^29–31^ pathway database as a source for the biological networks describing the studied phenomena. The regulation of autophagy pathway (KEGG ID: sce04140) describes the phenomena involved in sporulation. This pathway consists of mechanisms involved in processing internal and external stresses including nutrient availability. As a result, regulation of autophagy is essential for survival because it is used to maintain important cellular functions when environmental conditions change.

The QCD method was applied on the regulation of autophagy pathway and gene expression data from the yeast sporulation study by Chu *et al*. (GSE27,^21^). Panel A in Fig. 7 shows this pathway, as well as the genes measured in this experiment, marked in red. The experiment spanned 11.5 hours and data were collected at seven unequally spaced time points (0, 0.5, 2, 5, 7, 9, and 11.5 hours). The experiment was designed such that the sampling captures all known stages of the biological process. Sporulation is divided into four major stages: early, middle, mid-late, and late^21^.

The commitment to sporulation starts in the middle stage (2–5 h) and spans the mid-late stage (meiosis II phase, 5–7 h)^21^. Therefore, the true change interval for this phenomenon is 2 h to 7 h. As observed by Chu *et al*., the transition phase ends after the mid-late stage. This study also showed that one of the first discernible steps of spore morphogenesis occurs after the meiosis II spindles are formed, which makes the late phase a stable one. Also, the middle-late phase is still part of the change interval as previous studies reported that the middle-late phase includes the major cytological events of sporulation^32,33^. Panel B of Fig. 7 displays the measured changes of the genes on the regulation of autophagy pathway over the time course noted above.

In this case, QCD identifies a qualitative change in the interval from 0.5 h to 7 h, which includes the real change interval (2 h to 7 h) and starts one time point earlier. The change interval is the transition that separates the two potential meta-states (active state and spore state). The active and spore potential meta-states have p-values of $p=0.062$ and $p=0.0195$, respectively.

Sometimes small gene-level changes (not noticeable by eye) across the system can lead to important systemic changes. This is exactly the problem that our method was designed to address: the inability to easily identify important qualitative changes when they happen incrementally. The transition from healthy to disease is in many cases similar to the transition from young to old: any two consecutive measurements taken at short intervals are unlikely to show any important changes. However, the transition is happening and at some point, the current state will be significantly different from states long before. Our method is designed precisely for the purpose of detecting such changes and distinguishing them from mere random fluctuations present in any stable state.

### Fruit fly metamorphosis

Three major states — egg, larva and pupa — occur during the development of the fruit fly. The larvae typically pass through three molting stages (instars) during which they shed various body elements and form new ones. Importantly, the third molting stage the larvae pupate and become adults, which marks the completion of the metamorphosis process.

The QCD method was applied on the Hedgehog signaling pathway from KEGG^29–31^ (pathway ID: dme 04340) and data publicly available for the metamorphosis of *D. melanogaster* (GSE3057,^22^). The Hedgehog signaling pathway, named after the signaling molecule Hedgehog (Hh), has a crucial role in organizing the body plan for the fruit fly during development. Panel A in Fig. 8 shows this pathway as well as the genes measured in the metamorphosis experiment (in red in this figure). The experiment started 18 hours before pupariation, spanned 30 hours, and was sampled at nine time points, two prior to pupariation ($-18$ hours and $-4$ hours), and the other seven time points equally spaced over 12 hours after the actual pupariation (0 h, 2 h, 4 h, 6 h, 8 h, 10 h, 12 h).

Panel B of Fig. 8 shows the measured changes of the genes on this pathway over the time course described above. Puparium formation is triggered at the end of the third instar larvae stage that occurred during this experiment in the interval from $-4$ hours to 0 hours, and is marked by a high peak of the steroid hormone 20-hydroxyecdysone^22^. A second peak of the steroid hormone 20-hydroxyecdysone occurs roughly at the 10-hour time point and triggers the transformation from prepupa to pupa^22^. Puparium formation represents the onset of metamorphosis; therefore, the real change interval for this case study is indeed from $-4$ hours to 0 hours. The QCD method identifies one change interval from $-18$ hours to 0 hours. Notably, the third instar larvae stage, which starts 24 hours before pupariation and lasts until 0 hours (prepupae phase starts), is not a stable state in which the organism (fruit fly) exists. Therefore, the QCD not only correctly identifies the qualitative transition from larva to pupa, but it also shows the organism is in a continuous transition during the third instar larvae stage. The second change in this experiment (prepupa to pupa) arguably perturbs the system less than the first one since both prepupa and pupa are part of the pupal stage.

Notably, in this case study the change takes place at the beginning of the time course. To determine potential-meta-states relative to this change interval, we selected the only state before the change interval ($-18$ h) as the potential meta-state 1 and all states after the change interval (0 h–12 h) as potential meta-state 2. These two meta-states are characterized by highly significant p-values: $p=7.81\times 1{0}^{-3}$ and $p=3.73\times 1{0}^{-9}$, respectively.

### Fruit fly acute ethanol exposure

The fruit fly has been used as a model to study drug addiction. In the fruit fly, drug addiction produces physiological effects similar to those observed in mammals because the cellular neuronal mechanism that mediate the signals from the chemical compounds found in these drugs is conserved across these species.

To apply the QCD method, we used the Hedgehog signaling pathway (KEGG ID: dme04340) and the acute ethanol exposure data available from GEO (GSE18208) and described by Kong *et al*.^23^. The Hedgehog signaling pathway was chosen for its capability to model major mechanisms involved in fruit fly development, including its adaptive mechanisms. Panel A in Fig. 9 displays this pathway, as well as the genes measured in this experiment, marked in red. Panel B of Fig. 9 shows the measured changes of the genes on this pathway over the time course from the biological experiment. The experiment spanned 3.5 hours (210 minutes) of recovery after a 30-minute ethanol exposure, sedating up to 75% of the flies. Samples were taken at eight time points. The time points include one control, before exposure, one at 0 hour, right after exposure and every 30 minutes after that up to 3.5 hours; the missing data point at 2.5 hours (150 min) was not provided in the dataset. This experiment’s treatment conditions included exposure to humidified air or ethanol vapor (60%) for 30 minutes, and then recovery for up to 210 minutes^23^. The recovery period from ethanol sedation has been reported by another study to be approximately between 40 minutes and 2 hours^34^, which is the real change interval. Based on this recovery time, by the end of this experiment (210 minutes), the fruit flies should recover from the effects of ethanol exposure. In the GSE18208 dataset 40 minutes was not one of the sampled time points; therefore, to mark the real change interval, we used the very next time point available in the dataset, the one-hour time point.

The intuitive physiological transitions expected for these data are from no exposure (sober) to exposure to ethanol (drunk) and back to fully recovered (sober). However, the drunken state is temporary, since it is followed by recovery. Because of this transition, we expected two change intervals, from sober to drunk and from drunk to sober. Furthermore, the initial and end states (sober before exposure and sober after recovery) were expected to be very similar from a gene expression point of view. In other words, the sober state is the same in the initial and final state in this case, as opposed to the flagellum building case where the initial and final states, with and without flagellum, are obviously different.

The ethanol exposure has a delayed effect at the gene level. According to Kong *et al*., the expression of immunity genes increased after ethanol exposure in the time range from 0.5 hours to 1.5 hours^23^. Because of this delayed effect, we did not expect the biggest changes between the control and 0 hours but rather between the control and some later time point(s).

The QCD results on these data have shown that the biological system indeed goes through two qualitative changes, and the change intervals are: 0.5 hours to 1 hour and 1 hour to 1.5 hours, matching the expected transitions from a sober state to a drunken state and then back to the sober state. The effects of the ethanol exposure appear to peak at the 1-hour time point. Based on the change intervals and the return of the system to its initial state, there are two groups of states that may form meta-states. These potential meta-states consist of the following time points: control, 0 hour, 0.5 hours, and 1.5 hours to 3.5 hours, for meta-state 1, and the 1-hour time point for meta-state 2. The distribution of the significant and non-significant transitions yielded a highly significant p-value, $p=1.37\times 1{0}^{-5}$, for meta-state 1, but a non-significant p-value ($p=0.22$) for meta-state 2. This result is probably due to the small number of comparisons involving the single time point included in meta-state 2.

### Human hepatitis C virus (HCV) infection to hepatocellular carcinoma (HCC) progression

Hepatocellular carcinoma (HCC) is a common liver cancer that can be the result of an infection with the hepatitis C virus (HCV). The progression from HCV infection spans multiple disease stages before reaching HCC, as reported by Wurmbach *et al*.^35^. We used the data from this study to identify qualitative changes for this phenomenon. The dataset (GSE6764,^35^) contains gene expression collected from 75 samples (48 patients) and covers eight progressive stages of HCV induced HCC: four no-cancer stages including no HCV/control, cirrhosis, low-grade dysplastic, and high-grade dysplastic, and four cancer stages including very early HCC, early HCC, advanced HCC, and very advanced HCC. Normal liver control is used as the initial stage and stages are ordered by disease progression.

**Figure 10:**
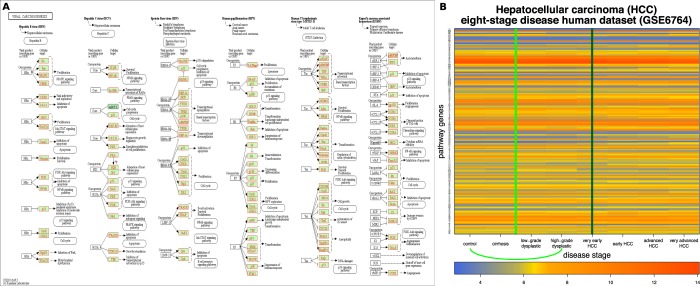
The input and results for QCD on human hepatitis C virus (HCV) to hepatocellular carcinoma (HCC) progression. The input is the viral carcinogenesis pathway from KEGG^29– 31^ (hsa05203), in Panel (A), and gene expression data from GEO GSE6764, in Panel (B). The data captures the progression from human HCV to HCC, specifically the transition from control (healthy) through the progressive stages of liver damage up very advanced HCC. Panel (B) shows the heatmap of the disease progression (control to very advanced HCC) for the measured KEGG pathway genes (in red), with the change interval detected for the phenomenon (green arc and the green line in the center of the interval (control – high-grade dysplastic nodules)). The dark green vertical line (very early HCC) marks the pre-disease state detected by the DNBM method).

To apply QCD on these data, we used the viral carcinogenesis pathway from KEGG^29–31^ (hsa05203) as the network/map of the biological system. The viral carcinogenesis pathway describes the signaling mechanisms involved in inflammatory responses such as the one triggered by HCV. Panel A in Fig. 10 shows this pathway as well as the genes measured in this experiment marked in red. Panel B of Fig. 10 shows the measured changes of the genes on this pathway over the different disease stages from the biological experiment.

From these data, the QCD identified one qualitative change (change interval) from stage zero (control), a benign state to stage three (high-grade dysplastic), the last of the four benign states and a state in which treatments are effective. The group of states before the change interval was considered as potential meta-state one (MS1) and contains only the control state. The group of states after the change interval was considered as potential meta-state two (MS2) and contains five states: high grade dysplastic nodules, very early HCC, early HCC, advanced HCC, and very advanced HCC. In essence, the analysis identified the transition from the benign state (first meta-state) to the cancerous state (second meta-state). The p-values of these meta-states were $p=0.031$ for MS1 and $p=3.05\times 1{0}^{-5}$ for MS2.

We compared the results of QCD in this case to the results of an existing method developed to detect network biomarkers and the pre-disease state (DNBM)^11^. The DNBM takes as input both the high-throughput data and the large network of protein-protein interactions for the organism under study. The output of DNBM is a pre-disease state in the form of a sample or list of samples from the data. The hypothesis is that a subset of the large network, termed the leading network, is the first to change toward the disease state, which makes its components and structure causally related with the disease. The DMBM models the change in gene expression over time as a Markov process. Then, a state-transition-based local network entropy (SNE) is used as a general, early measure of upcoming transitions by estimating the resilience of the network. The SNE is a Shannon-type entropy^36^, intended to quantify the change in state for the biological network.

Notably, the DNBM identifies one single (pre-disease) state prior to the onset of disease, while the proposed QCD identifies a change interval of transition to disease, which can be much more informative regarding the disease evolution, as well as providing an opportunity for therapeutic intervention. In addition, in the case of the QCD, the impact analysis approach may provide a better evaluation of the system’s impact than the network entropy. At the same time, a reinforcement of the impact by comparing every two time points may provide a better approximation of the change onset. Therefore, evaluating the systemic change between every two time points results in the early-detection property.

For this case study, the DNBM detected the pre-disease state at the fifth stage, very early HCC, which is the first malignant stage. The existent DNBM detected the start of the malignant state while our proposed QCD method detected the transition from benign to malignant.

DNBM was also evaluated on a dataset for mouse exposure to carbonyl chloride (phosgene). Exposure to carbonyl chloride produces irreversible lung injury and potentially life-threatening pulmonary edema that manifest within a day. We also evaluated the QCD on the same dataset (see Supplementary Methods Section 1.2 for details). The results in this case yielded perturbation factors that were hard to separate into large and small perturbations resulting in a poor fit of the mixture of gamma distributions. This is indicated by a larger value of the KLD and smaller value of the KS p-value. For this data set, the KLD yields a value of 2.92 (compared to the other data sets for which KLD values are around 0.1 or less). Also, the same dataset yields a KS p-value of 0.39. This is still far from being significant but also very significantly different from all the others which are above 0.85). Even in this case study, with the worst fit, the QCD method identified one qualitative change which corresponds to the time interval for the initiation of latent effects of the toxic gas exposure. In other words, QCD identified an interval during which damage is treatable^37^, while the DNBM identified a later time point as being the pre-disease state.

These results show the applicability of this method in developing preventive therapies. Identifying the genes that change within the change interval could lead to the identification of very early markers for disease and potential targets for disease prevention. A detailed description of the results of the QCD analysis at each step of the analysis workflow for all eight datasets is included in Section 1 of the Supplementary Methods.

In the case of disease progression, once a change interval is identified one should start the therapeutic intervention as early as possible within the change interval. For example, in the case of the HCV to HCC progression that could be any time up to the high-grade dysplastic stage.

To further evaluate the potential of the proposed method to detect changes as they occur, we ran the method on data from only the first three stages of the disease progression. DQC detected a change interval from the first (control) to the third stage (low-grade dysplastic), showing that a systemic qualitative change is happening and can be detected at a very early stage, as soon as the disease process has started.

## Discussion

Disease prevention and early detection are two major healthcare objectives that contribute to improving quality of life. Currently, early detection of complex diseases is achieved only after the physiological traits of the phenotype are present, when existing treatments may be ineffective. Chronic disease, a particular case of complex disease, is generally detected in the late stage of a relatively slow, progressive process. Representative examples that affect a large number of people are heart disease, cancer, and neurodegenerative disorders. It is a real challenge for people with these diseases to maintain a good quality of life after diagnosis. Understanding when the transition to disease occurs is a good first step towards interrupting the process and maintaining the healthy state.

To maintain the healthy state, one needs to monitor the biological system and measure the gene expression or any parameters the system has in order to assess how much the system is changing. The moment a qualitative change occurs, either cumulative or sudden, a change interval emerges. For instance, in the case of the eight stages of HCC, a qualitative change occurs from control to high-grade dysplasia. A cirrhotic liver is characterized by the presence of scar tissue due to long-term damage. In an attempt to replace the damaged cells in the cirrhotic liver, clusters of newly formed cells can occur in the scar tissue. Dysplastic (abnormally grown) nodules found in the liver are typically identified in cirrhotic livers. Low-grade dysplastic nodules (LGDN) cells are larger than the normal liver cells^38^. High-grade dysplastic nodules (HGDN) cells are smaller than the normal liver cells and have a greater nucleus-to-cytoplasm-size ratio^38^. The difference between HGDNs and very early HCC is the stromal invasion present in the latter^39^. A study on the LGDNs and HGDNs in HCC development concluded that LGDNs together with large regenerative nodules, should be monitored with ultrasound, while HGDNs should be preventively treated due to their high malignant risk^40^. Taken together, these data support the qualitative change identified by QCD from a low malignant risk stage of the liver disease to a high risk stage and close precursor to the malignant stage of very early HCC.

To further investigate the results of our analysis in the case of HCC progression, we identified the differentially expressed (DE) genes (absolute log${}_{2}$ fold change greater than 1) when comparing the control to high-grade dysplasia and the control to very advanced HCC. The total number of measured genes is 20,156. In the control versus high-grade dysplasia comparison, there are 149 DE genes, while in the control versus very advanced HCC comparison, there are 1,355 DE genes, which is almost an order of magnitude higher. This suggests that using the differentially expressed genes across the change interval, as opposed to the genes that differ between the control and very advanced HCC, offers a more focused analysis. In essence, the comparison across the narrowest change interval targets the genes involved in the initial tumor formation, rather than all genes that change as a consequence of the cancer.

The number of common DE genes among the two comparisons is 80, representing 53% of the initial 149 genes. We downloaded the curated list of cancer genes available in the cancer gene census^41^ (http://cancer.sanger.ac.uk/census). This list is presented together with the catalogue of somatic mutations in cancer (COSMIC)^42^ (http://cancer.sanger.ac.uk/cosmic). We used this list of cancer genes to filter the 80 common genes to obtain a cancer gene set. The result consists of two genes: *CHEK2* and *FAT1* (see Section 2.1 in Supplementary Methods for the expression profile). These genes are highly relevant to the condition under study considering *CHEK2* mutations have been linked to various cancers^43,44^; it has also been shown to be a mediator of a tumorigenic mechanism in HCC^45^. Furthermore, *FAT1* has been shown to have an oncogenic role in HCC^46,47^, and it has been identified as a biomarker in multiple cancers^48,49^.

The viral carcinogenesis pathway from KEGG^29–31^ was used to identify the change interval for the HCV-induced HCC progression. We also used this pathway to filter the 80 common genes and to obtain a “viral carcinogenesis” gene set, which contains genes from the pathway that change at the onset of the disease. The result consists of two early growth response genes: *EGR2* and *EGR3* (see Section 2.2 in Supplementary Methods for the expression profile). *EGR2* has been shown to be an apoptosis promoter gene^50^, which is downregulated by miRNAs in cancer^51,52^. *EGR3* has been shown to be involved in a number of cancers and in the regulation of the immune response^53–56^, and this gene has recently been linked to HCC when it was used to inhibit the growth of tumor cells^57^.

We designed and implemented an analytical method capable of detecting qualitative changes in the state of a biological system by monitoring its gene expression levels. This has been conducted with no training on previous examples, with no expert supervision, and with thresholds set using sound statistical criteria. The only hypothesis used here is that a qualitative change will involve enough pathway components to perturb the pathway in a significant way. The method requires a network of the system, which may limit its applicability. However, most biological systems do have associated networks. For instance, the KEGG pathway database includes about 200 signaling pathways for human, about 190 signaling pathways for mouse and about 190 signaling pathways for rat. Many such pathway databases exist: KEGG^29–31^, Reactome^58^, BioCarta^59^, NCI-PID^60^, WikiPathways^61^, and PANTHER^62^. The proposed method leverages this existing body of knowledge which is expected to grow in the future. In principle, these diagrams can be used to study how the system changes between states. However, most or all existing analysis methods would require an a priori definition of the states to be compared. Once these states are defined, a myriad of methods can be used to identify differentially expressed genes or pathways. One of the major contributions of the proposed method is that it can detect significant system changes without somebody having to define them a priori just by monitoring the system.

To evaluate the proposed method, we used both synthetic and real data. The cases used for validation cover a wide range of biological phenomena and model organisms as presented in the Results section (see Table 1 for a summary). Identifying a change interval implies recognizing the transition the system goes through from a state of relative equilibrium to another. The states of relative equilibrium the system transitions from are denoted here as meta-states and the transition as the change interval. Notably, in each case study, the system transitions between meta-states that are of great importance if we hypothesize that such transitions are infrequent and that a qualitative change is required for a system to undergo such transitions. We also assessed the statistical significance of the potential meta-states for each of the eight case studies. Results show that out of 16 putative meta-states, 13 are significant at a threshold of 5% (see Table 2).

**Table 1:** Summary of the results for the analysis of synthetic (simulation) and real data for various phenomena in model organisms. From left to right, the columns of the table show the organism, the phenomenon studied, the data source (simulation or GEO dataset), the duration of the simulation or experiment, the number of measurements, the time interval reported by the algorithm as including a qualitative change, and the actual time interval in which the phenomenon was simulated (first three rows) or actually took place (next five rows). *Denotes the results of the existing method^11^.

Results summary					
Organism	Phenomena	Data source	Time points (# of samples)	Detected change interval	Real change interval
*E. coli*	Flagella building	mathematical model	0–600 min (21) equally spaced	180–300 min	240–270 min
*B. subtilis*	Sporulation	mathematical model	0–600 min (21) equally spaced	210–240 min	210–240 min
*C. elegans*	Avoidance reflex	mathematical model	1–8 ms (8) equally spaced	4–5 ms	3–5 ms
*S. cerevisiae*	Sporulation	GSE27	0–11.5 h (7)	0.5–7 h	2–7 h
*D. melanogaster*	Pupariation	GSE3057	$-18$–12 h (9)	$-18$–0 h	$-4$–0 h
	Acute ethanol exposure	GSE18208	untreated 0 h treated 0–3.5 h (8)	0.5–1 h & 1–1.5 h	1–2 h
*M. musculus*	Carbonyl chloride exposure	GSE2565	0–72 h (9)	0.5–1 h	8 h*
*H. sapiens*	HCV induced HCC progression	GSE6764	8 stages (8)	control–high-grade dysplastic	very early HCC*

**Table 2:** Summary of the results establishing the significance of the meta-states. In each case-study two potential meta-states were identified, relative to the time-course data or sequential series of system states provided as input. Meta-state1 consists of the group of states before the change interval. Meta-state2 consists of the group of states after the change interval. For each case study and each potential meta-state, we calculate a statistic as the number of time-intervals with status consistent with the status assigned in the corresponding theoretical meta-state. Here, we show the p-values (one tail, greater) computed for this statistic as it follows a binomial distribution with a theoretical likelihood of success of 50%. From a total of 16 meta-states: 13 (without ${}^{\oslash }$) are significant at a threshold of 5%.

Meta-states results summary		
	p-value Meta-state I	p-value Meta-state II
*E coli* flagellum building	$5.44\times 1{0}^{-19}$	$3.61\times 1{0}^{-28}$
*B subtilis* sporulation	$2.31\times 1{0}^{-19}$	$6.23\times 1{0}^{-32}$
Worm avoidance reflex	$4.28\times 1{0}^{-4}$	$4.28\times 1{0}^{-4}$
Yeast sporulation	$0.06{2}^{\oslash }$	0.019
Fruit fly pupariation	$7.81\times 1{0}^{-3}$	$3.73\times 1{0}^{-9}$
Fruit fly alcohol exposure	$1.37\times 1{0}^{-5}$	$0.22{7}^{\oslash }$
Mouse carbonyl chloride exposure	$0.69{6}^{\oslash }$	$9.39\times 1{0}^{-4}$
Human HCV induced HCC progression	$0.031$	$3.05\times 1{0}^{-5}$

It is important to emphasize that the proposed method accomplishes two goals. First, the method identifies qualitative changes. These changes are identified based on the system perturbation factors. Second the methods also identifies meta-states, if they exist. A meta-state is a group of states that are very similar to each other. Sometimes, qualitative changes happen between meta-states and sometime qualitative changes happen without clear meta-states on both sides of the change. The p-values in Table 2 are used to test the hypothesis that a group of states form a meta-state. In each dataset and each meta-state, there is a single test and a single p-value. The fact that some p-values are not significant, simply means that the states on that side of the qualitative change are not very similar to each other and do not form a meta-state. For instance, in the case of the mice exposed to phosgene, before exposure, the individual expression values may involve many physiological differences. However, after exposure, the changes induced by the toxicant are higher than any normal physiological differences since they are associated with severe chemical trauma ultimately leading to death.

The proposed method was applied on a wide range of biological phenomena and was able to detect important transitions between system meta-states with high accuracy in the first six case studies having a known change interval: building a motility motor in *E. coli*, spore formation in *B. subtilis* and *S. cerevisiae*, backwards movement triggered by the nose touch in *C. elegans*, and both acute ethanol exposure and metamorphosis in *D. melanogaster*.

We also compared QCD to an existing method developed by Liu *et al*.^11^ for detecting the pre-disease state and network biomarkers on two datasets. These are two case studies where the phenomena are more complex. When analyzing the data for the exposure to the toxic gas phosgene in mice, QCD identified the cellular damage at an earlier time point, when treatment is still effective^37^.

When analyzing data for hepatitis C virus infection progression to hepatocellular carcinoma (HCC) in humans, QCD identified the transition from control to high-grade dysplasia. In this case, the existing method identified as the pre-disease state, i.e., the “very early HCC” stage, which can be interpreted as the start of the malignant state. Importantly, the change interval detected by QCD immediately precedes this pre-disease state detected by the existing method and marks the transition from benign to malignant. Intervention during this interval may prevent this transition and disease progression may be halted.

To summarize, we have evaluated the proposed method QCD on both synthetic (noise free) and real (noisy) data, on a total of eight case studies for six model organisms and one human dataset and the QCD identified the qualitative changes in each case. We have also used both time course data as well as disease stages as system states in our analyses, and QCD performed well for both types of data.

An immediate application for QCD could be to identify when the transition between different disease stages happens for other diseases. However, QCD is a versatile approach that can be applied to systemic states in different contexts (time course, disease progression, drug dose, BMI, age).

The QCD method can also be applied in the study of drug synergies and synthetic lethality where it could identify the time interval when one drug sensitizes the cell and the second drug has maximum efficacy in a time-dependent way. In turn, this could maximize the effect of combination therapies for various diseases. Another important application for the conceptual framework described herein is the prediction of obstetrical disease in early pregnancy, so interventions can mitigate or prevent the “great obstetrical syndromes” that are primarily observed during the third trimester of pregnancy^63^. In future work, we plan to use the QCD method to predict obstetrical disease based on transcriptomics, metabolomics, proteomics, lipidomics, and other data. A system state in the QCD framework can be any of, but not limited to, the following: a developmental stage, the response to a certain therapeutic dose, the stage of a disease, patients who share physiological traits or disease outcome. The analysis of time series expression data using QCD could potentially be used to decide the duration of adjuvant chemotherapy or disease recurrence. However, the most important application of this approach would imply a paradigm shift: one could use a QCD-like approach with the aim of identifying the departure from the healthy state instead of diagnosing the onset of disease.

## Methods

### Qualitative change detection (QCD) method

In this paper, we propose a paradigm shift: instead of detecting the onset of disease, we would like to be able to detect the departure from the healthy state. The qualitative change detection (QCD) analysis presented here is able to detect intervals when a biological system undergoes qualitative changes such as the transition from healthy to disease.

The workflow of the analysis (see Fig. 2) consists of the following steps:

1. Compare the status of the system between each pair of time points using an existing statistical method called pathway impact analysis (IA)^13– 16^ and assess the levels of perturbation;
2. Separate large and small inter-state perturbations using a gamma mixture model fitted to the system perturbation by an expectation maximization algorithm;
3. Calculate the change interval(s) as the narrowest disjunct interval(s) of large changes.

In step 1 the perturbation of the system between all pairs of system states is computed utilizing IA. First, sequential states are assigned to the chronologically ordered time points or disease progression stages when the data were sampled. We then compare all pairs of systems states using IA^13^, which was previously developed to evaluate the pathway impact when comparing two phenotypes; herein, we use it to calculate a system/pathway impact factor for each comparison of two system states (time points). The input of impact analysis includes the changes in expression between the two time-points for the measured genes, while the output will be a perturbation factor for the pathway. The result of this first step will be a list of time intervals (comparisons) with their computed pathway perturbation factor.

The pathway impact analysis takes as input signaling networks (pathways) and a list of genes with their respective changes between two states of a system (e.g. condition vs. control). In a typical signaling pathway, nodes represent genes or gene products and edges represent signals, such as activation or repression, directed from one node to another. The goal of IA is to identify the pathways significantly impacted in a given phenotype by analyzing all measured expression changes for all genes, as well as all of their interactions, as described by each pathway. This type of analysis incorporates two types of evidence, which taken together estimate the disruption on a pathway when comparing two phenotypes. The first type is evidence given by the perturbation analysis. The magnitude of expression change (log fold-change) and the pathway structure are used to compute a perturbation factor for each gene (Eq. (1)). For each of the pathways edges such as activation, activation through phosphorylation and inhibition/repression are used in the analysis with the respective values (1, 1, $-1$). All other edges have a value of 0. This is part of the implementation of the impact analysis^13–16^. The gene perturbation factors are summed up to the pathway level to account for the observed pathway perturbation.

1 $$PF(g)=\Delta E(g)+\sum _{u\in US(g)}{\beta }_{ug}\cdot \frac{PF(u)}{\#DS(u)}$$

 $PF(g)$ - perturbation factor for gene g

 $US(g)$ - set of genes directly upstream of g

 ${\beta }_{ug}$ - strength of interaction between u and g

 DS(g) - set of genes directly downstream of g

 $\Delta E(g)$ - log fold change in expression for g

 # - cardinality

For the perturbation analysis, we sum the absolute value of the gene perturbation factors (Eq. (2)) so that the up-regulation and down-regulation do not cancel each other.

2 $$PF(P)=\sum _{g\in P}| PF(g)| $$

 $PF(P)$ - perturbation factor for pathway P

 $| \cdot |$ - absolute value operator

We use the all-gene approach, without gene weights; therefore, since we do not select differentially expressed genes, the enrichment part cannot be computed. The pathway perturbation factors are positive values with 0 marking no perturbation — the higher the value, the larger the pathway perturbation. We work under the assumption that the pathway perturbation factors follow a gamma distribution with mode = 0 when the pathway is not perturbed.

**Figure 11:**
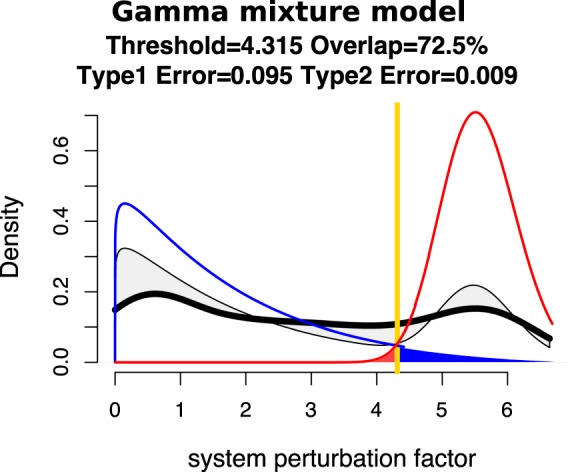
The fit of a mixture of two gamma distributions (blue and red lines) to the observed perturbation values of the system as computed for all pair-wise comparisons (thick black line). The fitted mixture distribution is marked by the thinner black line. The difference between the fitted and observed data is shaded in light gray. A goodness of fit measure is the overlap calculated as the ratio between the intersection and union of the areas under the observed data (thick black line) and fitted model (thinner black line). A perfect fit would yield an overlap of 100%. The null hypothesis is that there are no change intervals and therefore there are only small system perturbations (blue distribution). If a second distribution is found to be present (red), the threshold used to distinguish between small and large system perturbations will be the yellow vertical line. Under these circumstances, the blue area under the blue line is the Type 1 error and the red area under the red line is the Type 2 error.

In step 2, the distribution of the pathway perturbation factors is modeled using a gamma mixture model (see Fig. 11). The hypothesis states that if there is a change interval the system state comparisons will yield a mix of large and small system perturbations. Small system perturbations are expected when comparing system states before and after the change interval. Large system perturbations are expected when comparing system states before the change interval to states after the change interval. Therefore, a mixture of two gamma distributions is used: one for the comparisons in which the system is unperturbed (i.e., the null hypothesis) and another for comparisons in which the system is perturbed.

The EM algorithm has a number of parameters that can potentially influence the results. Such parameters include the initial shape and scale for the fitted gamma distributions, the convergence criteria (epsilon), the maximum number of iterations and the maximum number of restarts. The two gamma distributions parameters are initialized so that their modes corresponds to the minimum and maximum values of the perturbation factors, which puts the model in the correct range from the beginning. Other parameters such as the maximum number of iterations or epsilon are not influencing the results, as long as the values are reasonable. For instance, we use 100 as the maximum number of iterations but in all cases, the algorithm converged in fewer than 100 iterations. Therefore, even though in principle, the results can be influenced by the values of these various parameters, in practice the results were stable in all experiments we performed. In the proposed form, the user does not need to choose any parameters. As a potential improvement on the proposed technique, one could use a stochastic version of the EM algorithm.

The mixture model fitting will provide two distributions that best fit the data together with a percentage that estimates how much of the observed data comes from each of these two distributions. If any of the distributions has a percentage of less than 10%, the QCD analysis considers that there is only one distribution and, therefore, there is no significant change, and no change interval. The algorithm for this step is available in Supplementary Materials Section 1.1.

If both distributions fitted contribute more than 10%, the goodness of fit is then evaluated by computing the percentage of overlap between the observed and fitted distributions of system perturbations (see Fig. 11, overlap). Other statistical approaches (the Kolmogorov-Smirnov test and the Kullback-Leibler divergence) are also used to evaluate the goodness of fit and results are presented in the Supplementary Methods Section 1.3. If the mixture contains more than 10% of either of the distributions, the intersection of the two distributions is used as the threshold to select comparisons with large system perturbations. Comparisons that yield a pathway perturbation factor higher than this threshold will be marked as having a large system perturbation.

We also explored the alternative of using the fit of a single gamma distribution to the perturbation factors in order to decide if there is a systemic change captured by the data. In the case of fitting only one distribution for that purpose the results are slightly worse, or just as good in most cases (see Supplementary Methods Section 4).

An important requirement is to demonstrate that the approach does not report false positive changes in random data or in cases in which there are no changes in the organism. Section 3 in Supplementary Materials includes the results obtained with controls only, as well as results obtained with random data. These results show that the proposed approach does not report falsely significant changes.

In step 3, change intervals are computed as the overlap of comparisons with a large system perturbation using an algorithm based on the definition in the subsection titled “Change interval formal definition”. The algorithm takes as input a list of comparisons with an assigned system perturbation value and a predefined system perturbation threshold computed in step 2 described above. The algorithm iterates over the list of comparisons and identifies the start and end points of change intervals as points that have at least one comparison that shows a large perturbation (higher than the threshold) starting or ending in the respective points, and no large perturbation comparisons start or end in between those points. The output is a list of change intervals described by their start and end points. Note that the change interval does not have to be a comparison that shows a large system perturbation by itself.

**Change interval formal definition**

Notations:

 $N$ = the number of time points

 $S$ = the set of states

 $p$ = the set of perturbation values

 $CI$ = the set of change intervals

 $pcut$ = the perturbation threshold

Definitions:

 $$S$$ = $$\{{S}_{i}| i\in \{0,\ldots ,N-1\}\}$$

 $p$ = $\{{p}_{ij}| i,j\in \{0,\ldots ,N-1\},i < j\}$, where ${p}_{ij}$ is the system perturbation value when comparing ${S}_{i}$ and ${S}_{j}$

 $CI$ = $\{(x,y),x,y\in \{0,\ldots ,N-1\},x < y\}$, that satisfy the following conditions

 $$\forall i,j\in \{x,\ldots ,y\},(i,j)\ \ne \ (x,y)$$ and $${p}_{ij}\le pcut$$

 (i) $$\exists i\in \{0,\ldots ,y-1\}$$ such that $${p}_{iy} > pcut$$

 (ii) $\exists j\in \{x+1,\ldots ,N-1\}$ such that ${p}_{xj} > pcut$

 (iii) $x$ is the max value to satisfy the above conditions for a given $y$

 (iv) $y$ is the min value to satisfy the above conditions for a given $x$

### Meta-states statistical validation

To better understand the phenomenon under study, after the detection of a change interval, the states of the system before and after a change interval should be analyzed to gain insight regarding the state of the system before and after a qualitative change. To describe this analysis, the situation in which there is a single change interval will be considered, as in the *E. coli* flagellum building dataset. In this case, the system is considered to be stable before and after the change interval. In this context, we group the states in which the system is stable into meta-states. We define a meta-state as a group of consecutive states that satisfy the following two conditions:

1. All comparisons between states within a meta-state have a small system perturbation;
2. All comparisons between states from a meta-state to states outside the meta-state (excluding the states in the change interval) have a large system perturbation.

In the above, definition, the “small” and “large” perturbations, are defined based on the threshold between the two gamma distributions computed in the previous step and shown as the yellow line in Fig. 11.

Note that all comparisons between the states within a change interval and the meta-states immediately before and immediately after it may have a small system perturbation. This is because, during the change interval, the system is in transition between the two meta-states; therefore, its state during the transition is a mix of the two meta-states that may not be qualitatively different from either of them.

**Figure 12:**
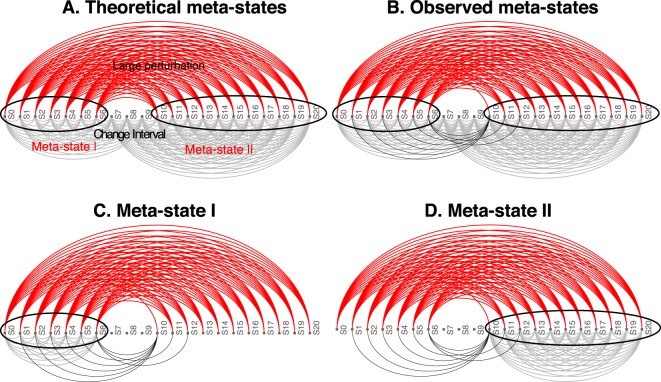
Meta-states in the *E. coli* flagellum building case study. Arc plots show possible comparison between time-points (states): comparisons with large system perturbation are red, and comparisons with small system perturbation are gray or black. Panel (A) the expected arc plots of two theoretical meta-states (groups of states in the black ellipses) relative to the detected change interval (S6–S10): all comparisons within each potential meta-state should show a small system perturbation while all comparisons between a meta-state time point and a time point outside the meta-state (excluding the change interval) should show a large system perturbation. Panel (B) the actual arc plot showing the observed large perturbation (red) vs small perturbation comparisons (gray and black) for all possible state comparisons. Black arcs show comparisons between states of potential meta-states (groups of states in the black ellipses) to states outside the potential meta-state (excluding the change interval) that show a small perturbation. Panel (C) the arc plot shows the observed comparisons for potential meta-state I (S0–S6, states in the black ellipse). Black arcs show comparisons between states of potential meta-state I to states outside it (excluding the change interval) that show a small perturbation. Panel (D) the arc plot shows the observed comparisons for potential meta-state II (S10–S20, states in the black ellipse). Black arcs show comparisons between states of potential meta-state II to states outside it (excluding the change interval) that show a small perturbation.

Based on the detected change interval, groups of sequential system states can form potential meta-states (see panel B in Fig. 12). Panel A in Fig. 12 shows the ideal results of all comparisons between all states involved in these meta-states. In essence, all comparisons within each potential meta-state should show a small system perturbation while all comparisons between a meta-state time point and a time point outside the meta-state (excluding the change interval) should show a large system perturbation.

To validate each observed potential meta-state, a statistical approach is applied to evaluate how closely it meets the conditions of a theoretical meta-state. The validation of the potential meta-state is described for the *E. coli* flagellum building dataset. The data was sampled at 21 time points (system states S0–S20) and the change interval was detected as (S6–S10). In this case, there are two potential meta-states: MS1, which contains the states before the change interval (states from S0 to S6), and MS2, which contains the states after the change interval (states from S10 to S20). To investigate the potential meta-states, all comparisons (arcs) (see Fig. 12B) are considered from the perspective of the meta-state definition above. For MS1, all comparisons between the states S0 to S6 should yield only small system perturbations. In addition, all comparisons between any states in MS1 and any states outside MS1 (not including the change interval) should involve large perturbations. With these considerations, all comparisons involving MS1 states can be assigned a binary value: either consistent or inconsistent with the expectations above. For each potential meta-state, a statistic is computed as the number of time intervals with status consistent with the status (large/small) assigned in the corresponding theoretical meta-state (meta-state definition). Under the null hypothesis, in which there are no groups of system states that form meta-states, the probability that a comparison is consistent or not should be 0.5. Based on this framework, a binomial model is used to calculate a p-value for the statistic computed for each of the groups of states that are potential meta-states (see Fig. 12C,D):

3 $$X \sim binom(n,{0.5})$$

 n - the number of trials;

 0.5 - probability that the status of a comparison

 is consistent with the meta-state definition.

The p-value computed for the potential meta-state characterizes the amount of evidence indicating the existence of a true meta-state (comparisons consistent with the definition, vs. inconsistent comparisons). A significant p-value lower than a predefined threshold would confirm the identification of a true meta-state. In our case studies, most p-values were significant at a 1% threshold (see details in Section 1.4 of the Supplementary Methods).

### Synthetic data parameters

For the *E. coli* flagellum building and *B. subtilis* sporulation, the gene expression synthetic data were generated using the interactions described by the biological network and Hill functions for protein accumulation (Eq. (4)) and decay (Eq. (5)) with a rate of $\alpha =0.005$.

 Given that $X\to Y$ denotes that transcription factor $X$ regulates gene $Y$

4 $$Y(t)={Y}_{st}{e}^{-\alpha \cdot t},{\rm{protein}}\ {\rm{accumulation}}$$

5 $$Y(t)={Y}_{st}(1-{e}^{-\alpha \cdot t}),{\rm{protein}}\ {\rm{decay}}$$

 ${Y}_{st}$ – steady state expression level for gene $Y$

 $\alpha$ – decay rate for protein $Y$

 $$t$$ – time

 $Y(t)$ – expression level for gene $Y$ at time $t$

For the third case study, *C. elegans*, data were generated using a step function for the ${X}_{1}=FLP$ neuron and a constant function (0) for the ${X}_{2}=ASH$ neuron. The following formula describes the change in voltage over time for the $Y=AVD$ neuron:

6 $$dY/dt=f(0.5\cdot {X}_{1}+0.5\cdot {X}_{2} > {K}_{Y})-Y$$

The following formula describes the change in voltage over time for the $Z=AVA$ neuron:

7 $$dZ/dt=f(0.5\cdot ({X}_{1}+{X}_{2})+0.4\cdot Y > {K}_{Z})-Z$$

Constants 0.5 and 0.4 are the strengths of the synaptic connections, and ${K}_{Y}$ and ${K}_{Z}$ are the activation thresholds.

For gene expression from biological experiments, microarray data were downloaded from the GEO database. The CEL files downloaded from GEO were processed using custom R scripts (R version 3.1.2). Data pre-processing (background correction and normalization) was performed using the threestep function from the affyPLM (version 1.42.0) R package. Gene IDs were mapped to gene symbols using the respective annotation packages from R: org.Sc.sgd.db (yeast), org.Dm.eg.db (fruit fly), org.Mm.eg.db and moe430a.db (mouse), org.Hs.eg.db and hgu133plus2.db (human). Gene expression at a specific time-point was computed as the average of the replicates for the specific time point when replicates were available. The ROntoTools 1.6.1 R package was used for impact analysis. The mixtools 1.0.3 R package was used for the mixture model analysis.

## Supplementary information

Supplementary information
