# Supplementary material for: Detecting qualitative changes in biological systems
Source: Sci Rep. 2020 May 18;10:8146. doi: 10.1038/s41598-020-62578-8 (PMC7235093; doi:10.1038/s41598-020-62578-8)
Supplement: Supplementary file 1 — Supplementary information [file 41598_2020_62578_MOESM1_ESM.pdf]

Detecting Qualitative Changes in Biological Systems
Supplementary Material

April 20, 2020

Cristina Mitrea[1,2], Aliccia Bollig-Fischer[2,3], Călin Voichiţa[1], Michele Donato[4], Roberto Romero[5,6,7,8], and Sorin Drăghici[1,9,*]

[1]Department of Computer Science, Wayne State University, Detroit, MI, USA.
[2]Department of Oncology, Wayne State University, Detroit, MI, USA.
[3]Karmanos Cancer Institute, Detroit, MI, USA.
[4]Institute for Immunity, Transplantation and Infection, Stanford University, Stanford, CA, USA.
[5]Perinatology Research Branch, Division of Obstetrics and Maternal-Fetal Medicine, Division of Intramural Research, Eunice Kennedy Shriver National Institute of Child Health and Human Development, NICHD/NIH/DHHS, Bethesda, MD, and Detroit, MI, USA.
[6]Department of Obstetrics and Gynecology, University of Michigan, Ann Arbor, MI, USA.
[7]Department of Epidemiology and Biostatistics, Michigan State University, East Lansing, MI, USA.
[8]Center for Molecular Medicine and Genetics, Wayne State University, Detroit, MI, USA.
[9]Department of Obstetrics and Gynecology, Wayne State University, Detroit, MI, USA.

1 QCD analysis results and the corresponding meta-states

The workflow of the QCD analysis consists of the following three steps: (i) compare the status of the system between each pair of time points using a pathway impact analysis [1, 2, 3, 4] and assess the levels of perturbation by computing a system perturbation factor; (ii) separate large and small inter-state perturbations (see Algorithm 1) using a gamma mixture model fitted to the system perturbation by an expectation maximization algorithm; (iii) calculate the change interval(s) as the narrowest disjunct interval(s) of large changes. Figures 2 - 9 show the results at each of these steps when applying QCD on eight case studies.

1.1 Algorithm for Identifying large and small perturbations

Data: p - List of pathway perturbation factors for each pair-wise comparison of time-points
Result: pcut - Threshold to separate p into large and small perturbations, or report NO CHANGE
initialization of G1 mode with min(p);
initialization of G2 mode with max(p);
epsilon = 10^{-8};
maxit =100;
iterations = 0;
pcut = 0;
diff = change in the observed data log-likelihood between the mix of G1 and G2 and p;
while *iterations* $\leq$ *maxit AND diff* $>$ *epsilon* **do**
  adjust shape, scale and percentage for G1 and G2 ;
  diff = change in the observed data log-likelihood between the mix of G1 and G2 and p;
  iterations = iterations +1;
end
if *percentage of G1* $\leq$ *0.1 OR percentage of G2* $\leq$ *0.1* **then**
  report NO CHANGE;
else
  pcut = intersection of G1 and G2;
end

Algorithm 1: Identifying large and small perturbations.

1.2 Details of the mouse exposure to phosgene case study

Carbonyl chloride (phosgene) is a toxic compound used for the production of materials such as plastics and rubber. Exposure to carbonyl chloride produces irreversible lung injury and potentially life-threatening pulmonary edema that manifest within a day. Early intervention, within one hour of exposure, has been reported to be effective for the treatment of carbonyl chloride exposure [5]. However, the damage inflicted by exposure to carbonyl chloride is progressive with the most significant physiological effects reported to occur between four and 12 hours after exposure [6]. Due to the high toxicity of carbonyl chloride, the organism will not return to its pre-exposure like state, yet it will be in a different state (injured or most likely lethally injured) state at 72 hours after exposure. In mice, by the 12th hour after exposure, a mortality rate of 50-60% was reported, which increased to 60-70% by the 24th hour [7].

In the study by Sciuto et al. [7], mice were exposed to 32 mg of phosgene per cubic meter for 20 minutes and samples were collected from lung tissue at nine time points: untreated (0 hours), 30 minutes, 1 hour, and 4, 8, 12, 24, 48, and 72 hours after exposure. We applied QCD to study this phenomenon, using these data, and the chemokine signaling pathway from KEGG [8, 9, 10] (mmu04062) as the network/map of the biological system. The chemokine signaling pathway was chosen because it describes the signaling mechanisms of an inflammatory response and such mechanisms are intimately involved in the response to the exposure to a toxic gas.

Panel A in Fig. 1 shows the chemokine signaling pathway as well as the measured genes marked in red. Panel B of Fig. 1 shows the measured changes of the genes on this pathway over the time course of the biological experiment. The QCD method identified one qualitative change in the interval of 0.5 hours to 1 hour which corresponds to the time interval for the initiation of latent effects of the toxic gas exposure. In other words, QCD identified an interval during which damage is treatable [5].

The change interval identified by QCD was then used to group the states (time points) before (0 hour to 0.5 hours) and after (1 hour to 72 hours) into potential meta-states. These groups of states can be labeled as "before exposure" and "long-lasting damage" based on the organism's physiology for the respective states. The groups of states were then evaluated for statistical significance and the p-value of the "before exposure" group was not significant ($p = 0.696$), while the "long-lasting damage" group had a highly significant p-value ($p = 9.39 \times 10^{-4}$), which makes it a true meta-state. This result may suggest that the control (no exposure)

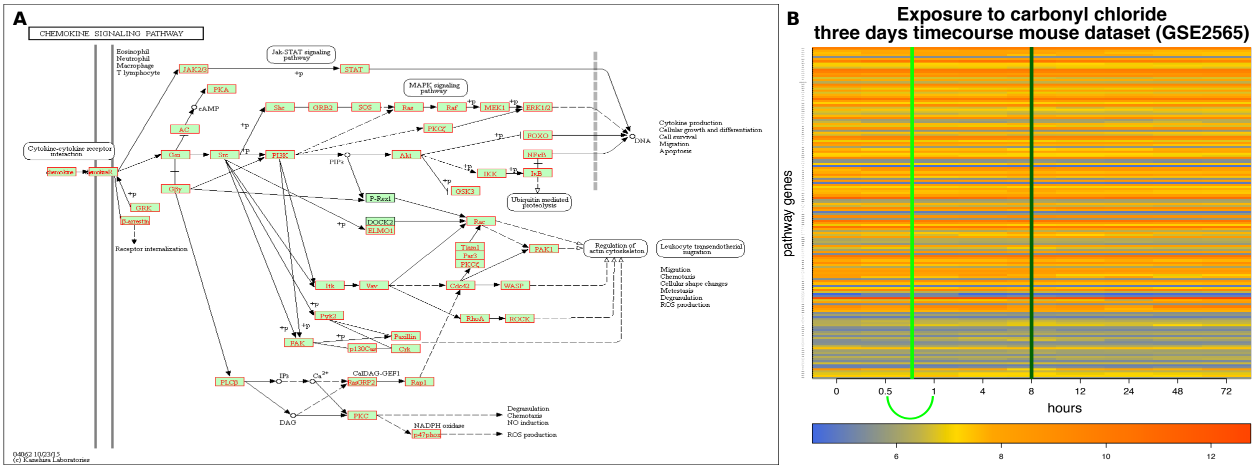

Figure 1: The input and results for QCD on mouse toxic gas exposure. The input is the chemokine signaling pathway from KEGG [8, 9, 10] (mmu04062), in Panel A, and gene expression data from GEO GSE2565, in Panel B. The data captures the exposure to phosgene phenomenon, specifically the transition from not exposed through the progressive damage of the exposure up to lethality. Panel B shows the heatmap of the time course (0 to 72 hours) for the measured KEGG pathway genes (in red), with the change interval detected for the phenomenon (green arc and the green line in the center of the interval ($0.5 - 1$h)). The dark green vertical line (8h) marks the pre-disease state detected by the DNBM method).

and the 30-minute after exposure system states are not similar enough to yield a statistically significant meta-state. This is very likely to be true since there is still damage inflicted at the 30-minute time point even though it is still treatable.

We compared the results of QCD in this case to the results of an existing method developed to detect network biomarkers and the pre-disease state (DNBM) [11], which identified the pre-disease state at hour 8.

1.3 Assessing the goodness of fit

After the second step (fitting the gamma mixture model), we compute several statistics to evaluate the goodness of fit between the observed perturbation factors and the fitted mixture model. In Panels A in Figures 2 − 9, KLD is the Kullback-Leibler divergence computed between the density of the observed perturbation and the density of the fitted mixture model. The KLD is a non symmetrical measure, therefore we compute both the KLD_o, o-observed first, and the KLD_f, f-fitted first, values. Values closer to 0 indicate higher similarity between the distributions. Our results show 7 out of 8 cases with KLDs less than 0.2. We used the KLD method from the R package LaplacesDemon 16.0.1 to compute this measure.

The KS_p is the p-value of the Kolmogorov-Smirnov test between the observed perturbation values and a sample of 10,000 values from the fitted mixture model. The null hypothesis in this case is that the samples come from the same distribution. A high p-value (close to 1), tells us that there is no evidence that the two distributions are significantly different. For instance, in the *E. coli* case, the p-value of 0.42 indicates that there is no significant difference between the observed distribution and the fitted one. Typical thresholds for rejecting the null hypothesis are 0.01, 0.05 and 0.1, and our results show p-values higher than 0.39, with 6 out of 8 values higher than 0.8. The ks.test method from the R package stats 3.1.2 was used to compute these p-values.

Another measure of the goodness of fit is the ratio between the intersection and union of the areas delimited by the observed and fitted density lines. We refer to this ratio as the overlap between the observed and fitted distributions. An overlap of 100% would mean a perfect match. The minimum overlap value on our case studies is 68.97% and the maximum is 89.5%.

In Panels A in Figures 2 − 9, Type 1 and Type 2 errors are computed under the null hypothesis when there are no change intervals and all system perturbations are small system perturbations. The lower the type 1 and type 2 errors the more reliable the results. The maximum type 1 error is 0.18, and the minimum 0.012 with most of them (5/8) case studies having a less than 0.1 type 1 error. The maximum type 2 error is 0.067, and the minimum 0.009 with all case studies having a less than 0.1 type 2 error.

1.4 Identifying meta-states

The states of the system before and after a change interval should be analyzed to gain insight regarding the state of the system before and after a qualitative change. To describe this analysis, we will consider the situation in which there is a single change interval, as in the *E. coli* flagellum building data set. We consider that in this case the system is stable before and after the change interval. In this context, we group the states in which the system is stable into meta-states. We define a meta-state (see Fig. 2, panel C) as a group of consecutive states that satisfy the following two conditions, using a system perturbation threshold previously computed: (i) all comparisons between states within a meta-state have a small system perturbation; (ii) all comparison between states from a meta-state to states outside it (excluding the states in the change interval) have a large system perturbation.

For any given change interval, we consider the groups of states before and after the change interval as potential meta-state. However, not every group of states before and after a change interval has to form a meta-state. Given a potential meta-state, any individual comparison between two individual states can be either consistent or inconsistent with the definition above. Under the null hypothesis, in which there is no meta-state, the probability that a comparison is consistent or not should be 0.5. This a priori probability of consistent/not consistent comparison was verified by a number of simulations (100,000) with random data where the mean and median of this probability were 0.501 and 0.5013, respectively. A large number of comparisons consistent with the definition of a meta-state will constitute evidence for the existence of such meta-state. A binomial distribution can be used for each meta-state to compute a p-value from the observed number of consistent comparisons for the given meta-state. From a total of 16 meta-states (2 for each of the

8 data sets included here), 11 were significant at a threshold of 1%, two were significant at 5%, and one at the 10% significance level. This suggests that most of the time, the organism transitions from a stable state to another stable state. Groups of states that do not form statistically significant meta-states may be due to a phenomenon that is still evolving, or simply to a low number of time points, which reduces the number of comparisons available, and therefore the statistical power of the test employed. An example of the latter situation is the ethanol exposure experiment in which the only meta-state eligible for consideration included a single time point (S3).

Figures 2 – 9 show the potential meta-states with their ideal comparisons according to the definition (panel C in each figure), as well as the observed comparisons (panel D in each figure). Panels E and F in Figures 3 – 9 show the meta-states considered, together with their respective p-values.

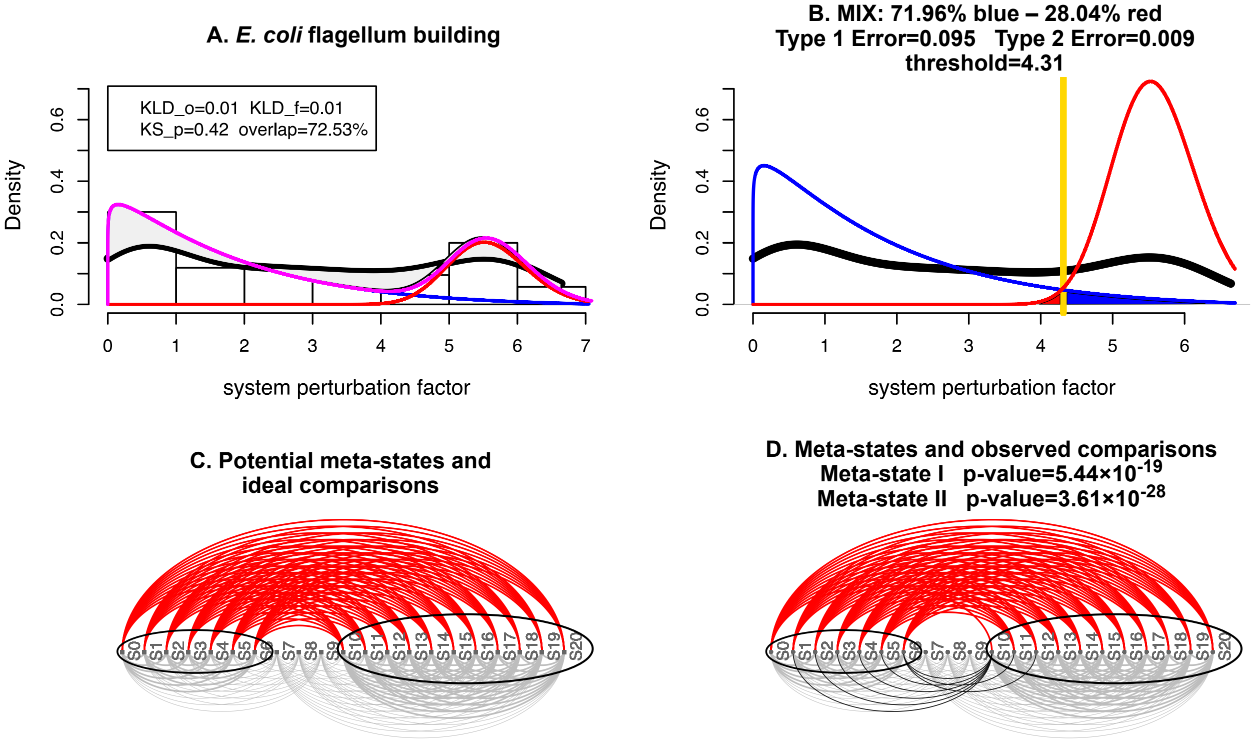

Figure 2: The results of QCD on synthetic data of the *E. coli* flagellum building. Panel A shows the mixture (magenta line) of two gamma distributions (blue and red lines) that is fitted to the perturbation factors (histogram, and density - thick black line). The blue and red density lines are scaled using the mixture proportion. We evaluate the goodness of fit using three statistics: (i) the Kullback-Leibler divergence between the density of the observed perturbation and the density of the fitted mixture model (KLD_o-observed first, KLD_f-fitted first); (ii) the p-value of the Kolmogorov-Smirnov test (KS_p) between the observed perturbation and the fitted mixture model; and (iii) the overlap, which is the ratio between the intersection and union of the areas delimited by the observed (thick black) and fitted (magenta) density lines. Panel B shows the gamma mixture model used to separate small (blue line) and large perturbations (red line). The blue and red distributions which compose the mixture model are unscaled in this panel and the mixture proportion is reported. The yellow vertical line is the threshold used to separate the small and large perturbation factors. The null hypothesis is that there are no change intervals and therefore there are only small system perturbations (blue distribution). The Type 1 and Type 2 errors are marked by the blue and red areas, respectively. Panel C shows the potential meta-states (black ellipses) together with the ideal comparisons between the time points within these meta-states (red - large perturbation, gray - small perturbation). Panel D shows the same meta-states (black ellipses) together with the observed comparisons (red - large perturbation, gray and black - small perturbation). Black comparisons are between states from different meta-states (these are red in the ideal case).

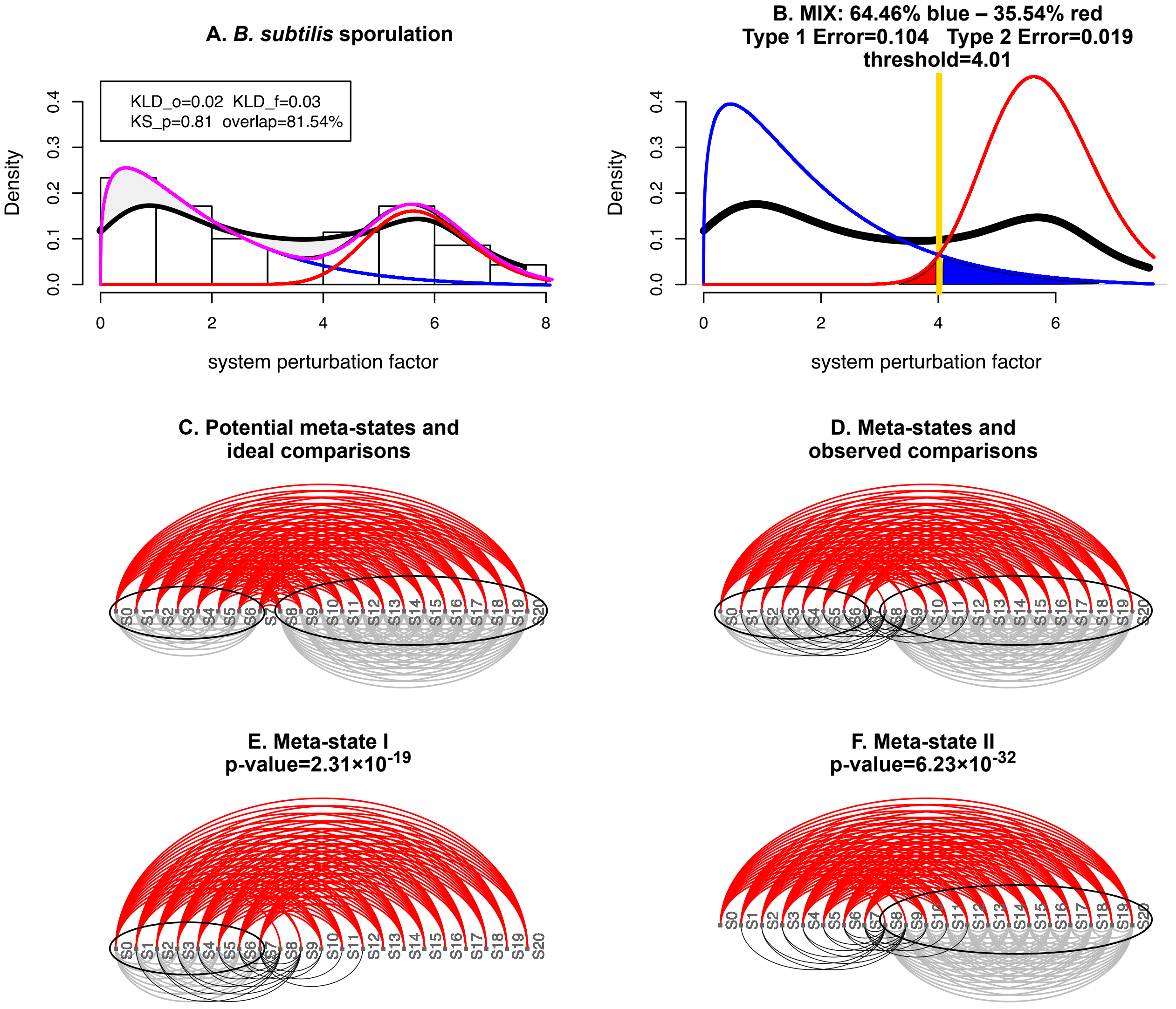

Figure 3: The results of QCD on synthetic data of the *B. subtilis* sporulation. Panel A shows the mixture (magenta line) of two gamma distributions (blue and red lines) that is fitted to the perturbation factors (histogram, and density - thick black line). The blue and red density lines are scaled using the mixture proportion. We evaluate the goodness of fit using three statistics: (i) the Kullback-Leibler divergence between the density of the observed perturbation and the density of the fitted mixture model (KLD_o-observed first, KLD_f-fitted first); (ii) the p-value of the Kolmogorov-Smirnov test (KS_p) between the observed perturbation and the fitted mixture model; and (iii) the overlap, which is the ratio between the intersection and union of the areas delimited by the observed (thick black) and fitted (magenta) density lines. Panel B shows the gamma mixture model used to separate small (blue line) and large perturbations (red line). The blue and red distributions which compose the mixture model are unscaled in this panel and the mixture proportion is reported. The yellow vertical line is the threshold used to separate the small and large perturbation factors. The null hypothesis is that there are no change intervals and therefore there are only small system perturbations (blue distribution). The Type 1 and Type 2 errors are marked by the blue and red areas, respectively. Panel C shows the potential meta-states (black ellipses) together with the ideal comparisons between the time points within these meta-states (red - large perturbation, gray - small perturbation). Panel D shows the same meta-states (black ellipses) together with the observed comparisons (red - large perturbation, gray and black - small perturbation). Black comparisons are between states from different meta-states (these are red in the ideal case). Panel E shows the comparisons considered for meta-state I. Panel F shows the comparisons considered for meta-state II.

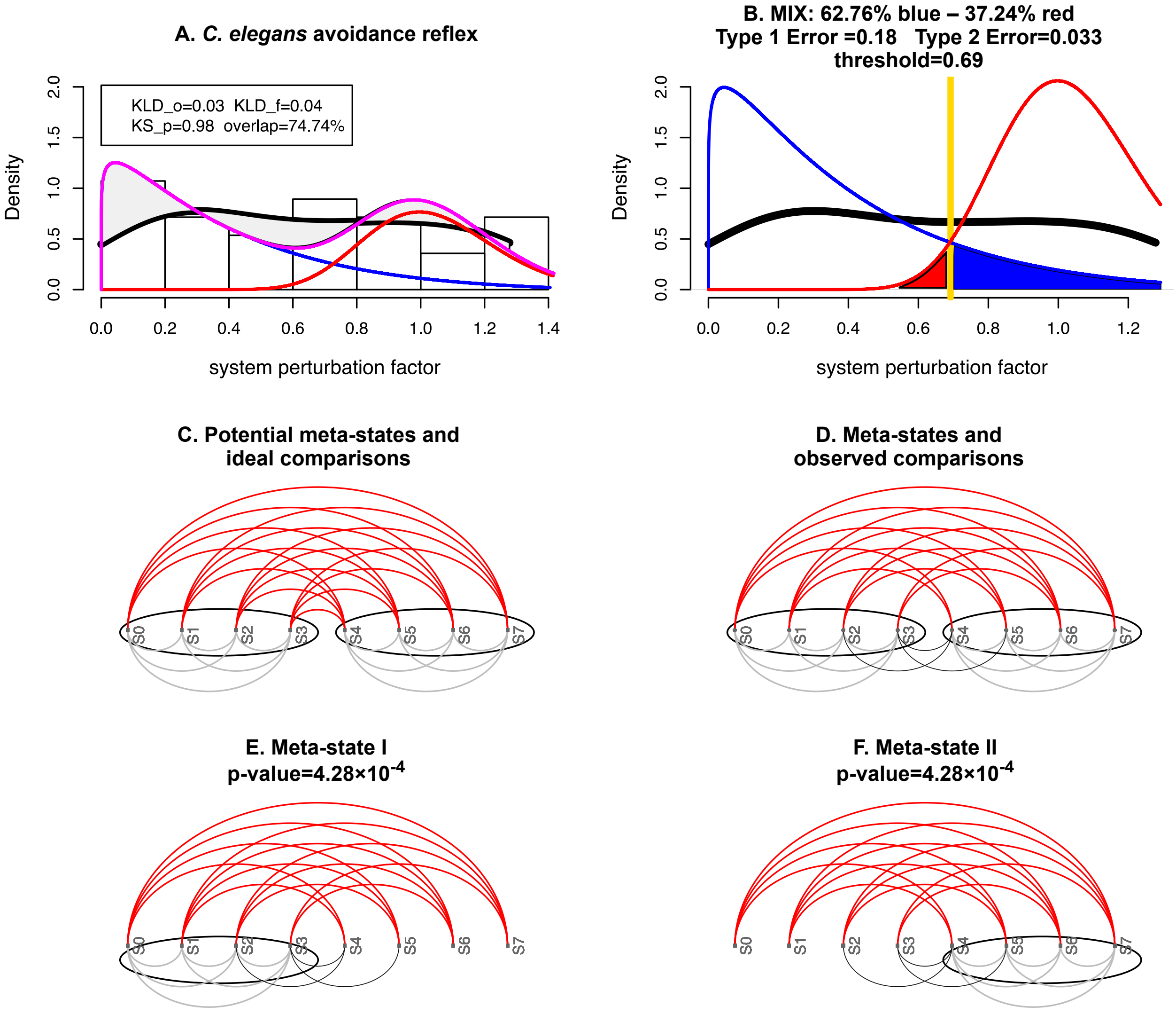

Figure 4: The results of QCD on synthetic data of the *C. elegans* avoidance reflex. Panel A shows the mixture (magenta line) of two gamma distributions (blue and red lines) that is fitted to the perturbation factors (histogram, and density - thick black line). The blue and red density lines are scaled using the mixture proportion. We evaluate the goodness of fit using three statistics: (i) the Kullback-Leibler divergence between the density of the observed perturbation and the density of the fitted mixture model (KLD_o-observed first, KLD_f-fitted first); (ii) the p-value of the Kolmogorov-Smirnov test (KS_p) between the observed perturbation and the fitted mixture model; and (iii) the overlap, which is the ratio between the intersection and union of the areas delimited by the observed (thick black) and fitted (magenta) density lines. Panel B shows the gamma mixture model used to separate small (blue line) and large perturbations (red line). The blue and red distributions which compose the mixture model are unscaled in this panel and the mixture proportion is reported. The yellow vertical line is the threshold used to separate the small and large perturbation factors. The null hypothesis is that there are no change intervals and therefore there are only small system perturbations (blue distribution). The Type 1 and Type 2 errors are marked by the blue and red areas, respectively. Panel C shows the potential meta-states (black ellipses) together with the ideal comparisons between the time points within these meta-states (red - large perturbation, gray - small perturbation). Panel D shows the same meta-states (black ellipses) together with the observed comparisons (red - large perturbation, gray and black - small perturbation). Black comparisons are between states from different meta-states (these are red in the ideal case). Panel E shows the comparisons considered for meta-state I. Panel F shows the comparisons considered for meta-state II.

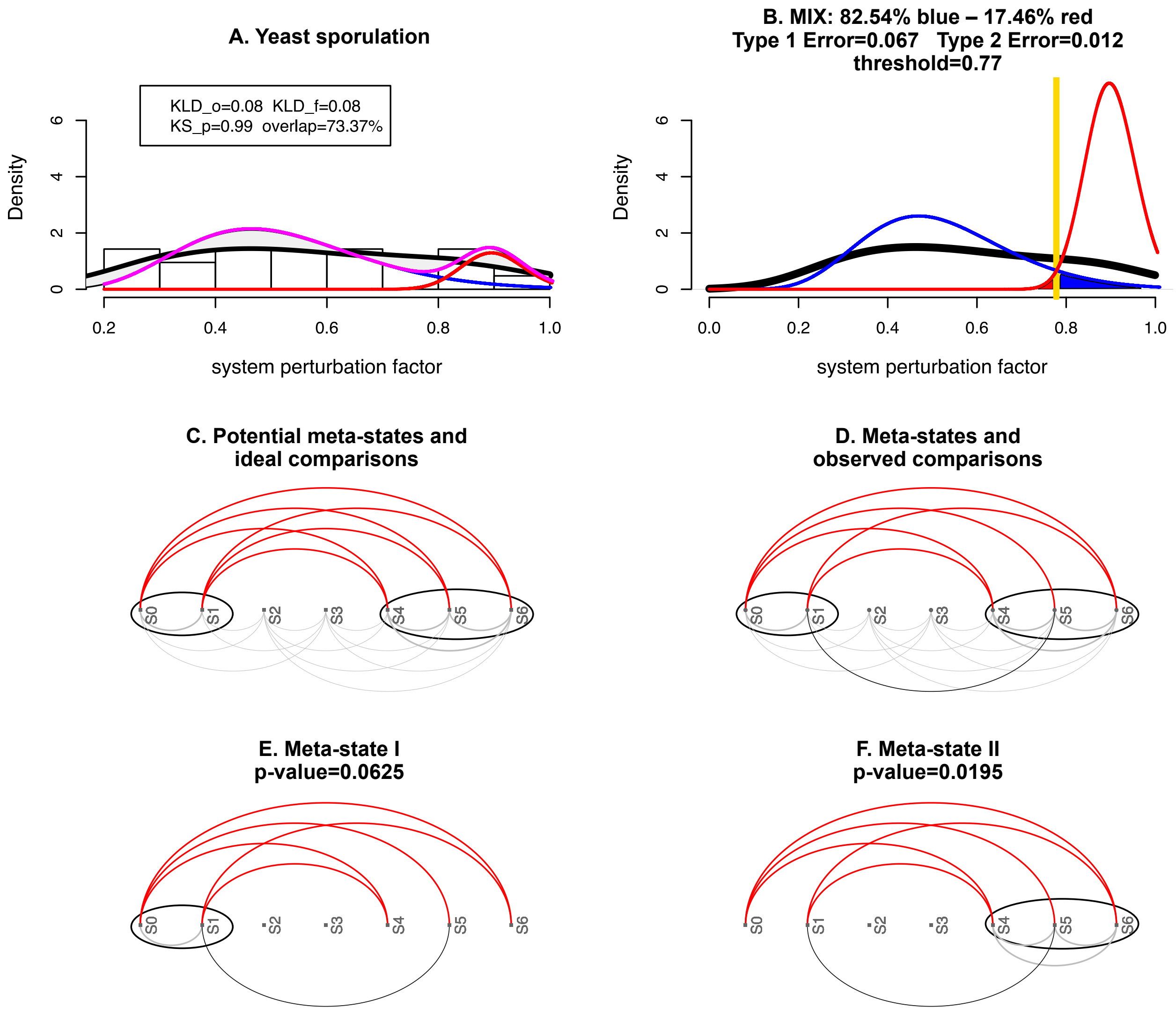

Figure 5: The results of QCD on real data of the yeast sporulation. Panel A shows the mixture (magenta line) of two gamma distributions (blue and red lines) that is fitted to the perturbation factors (histogram, and density - thick black line). The blue and red density lines are scaled using the mixture proportion. We evaluate the goodness of fit using three statistics: (i) the Kullback-Leibler divergence between the density of the observed perturbation and the density of the fitted mixture model (KLD_o-observed first, KLD_f-fitted first); (ii) the p-value of the Kolmogorov-Smirnov test (KS_p) between the observed perturbation and the fitted mixture model; and (iii) the overlap, which is the ratio between the intersection and union of the areas delimited by the observed (thick black) and fitted (magenta) density lines. Panel B shows the gamma mixture model used to separate small (blue line) and large perturbations (red line). The blue and red distributions which compose the mixture model are unscaled in this panel and the mixture proportion is reported. The yellow vertical line is the threshold used to separate the small and large perturbation factors. The null hypothesis is that there are no change intervals and therefore there are only small system perturbations (blue distribution). The Type 1 and Type 2 errors are marked by the blue and red areas, respectively. Panel C shows the potential meta-states (black ellipses) together with the ideal comparisons between the time points within these meta-states (red - large perturbation, gray - small perturbation). Panel D shows the same meta-states (black ellipses) together with the observed comparisons (red - large perturbation, gray and black - small perturbation). Black comparisons are between states from different meta-states (these are red in the ideal case). Panel E shows the comparisons considered for meta-state I. Panel F shows the comparisons considered for meta-state II.

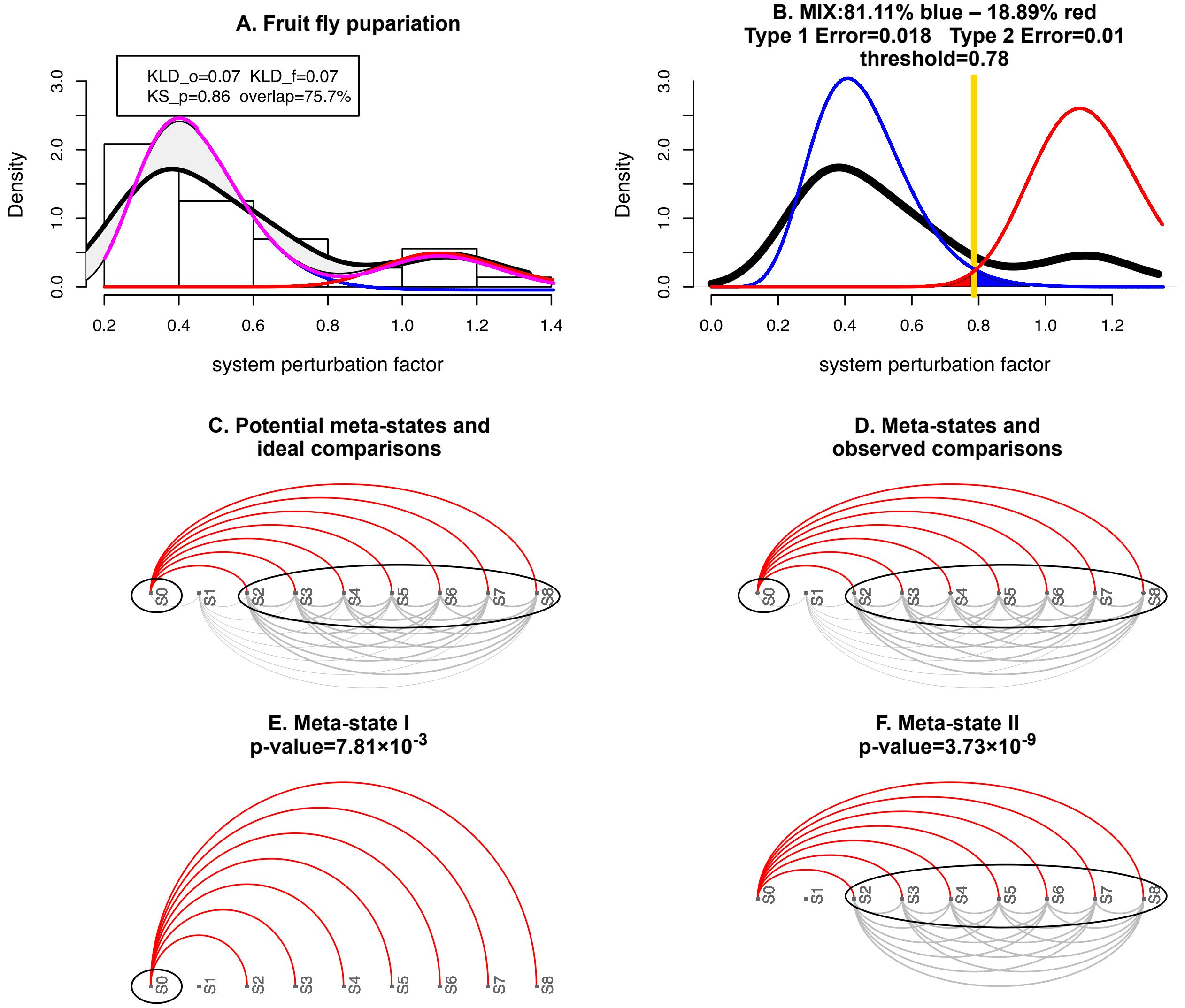

Figure 6: The results of QCD on real data of the fruit fly pupariation. Panel A shows the mixture (magenta line) of two gamma distributions (blue and red lines) that is fitted to the perturbation factors (histogram, and density - thick black line). The blue and red density lines are scaled using the mixture proportion. We evaluate the goodness of fit using three statistics: (i) the Kullback-Leibler divergence between the density of the observed perturbation and the density of the fitted mixture model (KLD_o-observed first, KLD_f-fitted first); (ii) the p-value of the Kolmogorov-Smirnov test (KS_p) between the observed perturbation and the fitted mixture model; and (iii) the overlap, which is the ratio between the intersection and union of the areas delimited by the observed (thick black) and fitted (magenta) density lines. Panel B shows the gamma mixture model used to separate small (blue line) and large perturbations (red line). The blue and red distributions which compose the mixture model are unscaled in this panel and the mixture proportion is reported. The yellow vertical line is the threshold used to separate the small and large perturbation factors. The null hypothesis is that there are no change intervals and therefore there are only small system perturbations (blue distribution). The Type 1 and Type 2 errors are marked by the blue and red areas, respectively. Panel C shows the potential meta-states (black ellipses) together with the ideal comparisons between the time points within these meta-states (red - large perturbation, gray - small perturbation). Panel D shows the same meta-states (black ellipses) together with the observed comparisons (red - large perturbation, gray and black - small perturbation). Black comparisons are between states from different meta-states (these are red in the ideal case). Panel E shows the comparisons considered for meta-state I. Panel F shows the comparisons considered for meta-state II.

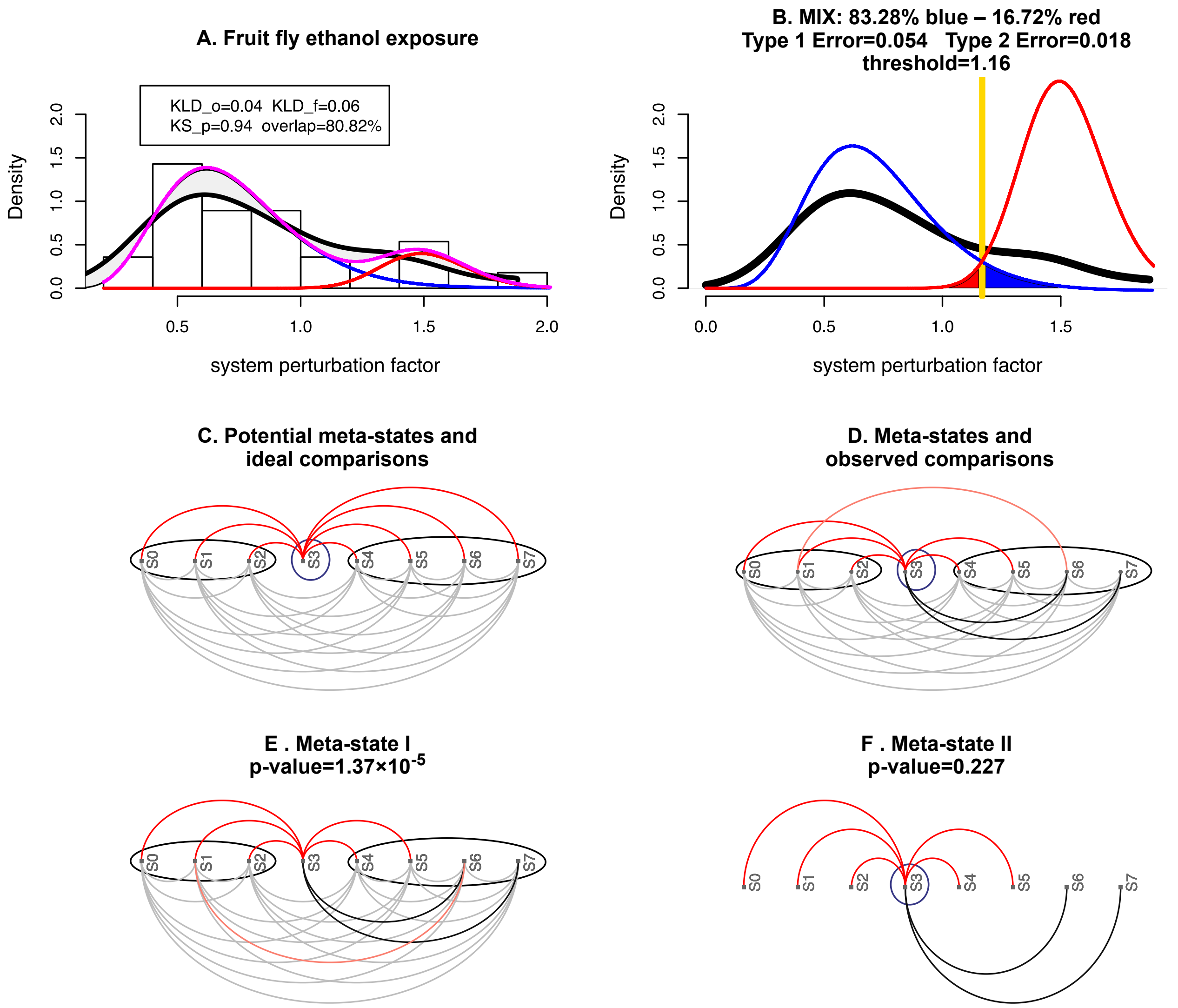

Figure 7: The results of QCD on real data of the fruit fly ethanol exposure. Panel A shows the mixture (magenta line) of two gamma distributions (blue and red lines) that is fitted to the perturbation factors (histogram, and density - thick black line). The blue and red density lines are scaled using the mixture proportion. We evaluate the goodness of fit using three statistics: (i) the Kullback-Leibler divergence between the density of the observed perturbation and the density of the fitted mixture model (KLD_o-observed first, KLD_f-fitted first); (ii) the p-value of the Kolmogorov-Smirnov test (KS_p) between the observed perturbation and the fitted mixture model; and (iii) the overlap, which is the ratio between the intersection and union of the areas delimited by the observed (thick black) and fitted (magenta) density lines. Panel B shows the gamma mixture model used to separate small (blue line) and large perturbations (red line). The blue and red distributions which compose the mixture model are unscaled in this panel and the mixture proportion is reported. The yellow vertical line is the threshold used to separate the small and large perturbation factors. The null hypothesis is that there are no change intervals and therefore there are only small system perturbations (blue distribution). The Type 1 and Type 2 errors are marked by the blue and red areas, respectively. Panel C shows the potential meta-states (black ellipses) together with the ideal comparisons between the time points within these meta-states (red - large perturbation, gray - small perturbation). Panel D shows the same meta-states (black ellipses) together with the observed comparisons (red - large perturbation, gray and black - small perturbation). Black comparisons are between states from different meta-states (these are red in the ideal case). Panel E shows the comparisons considered for meta-state I. Panel F shows the comparisons considered for meta-state II.

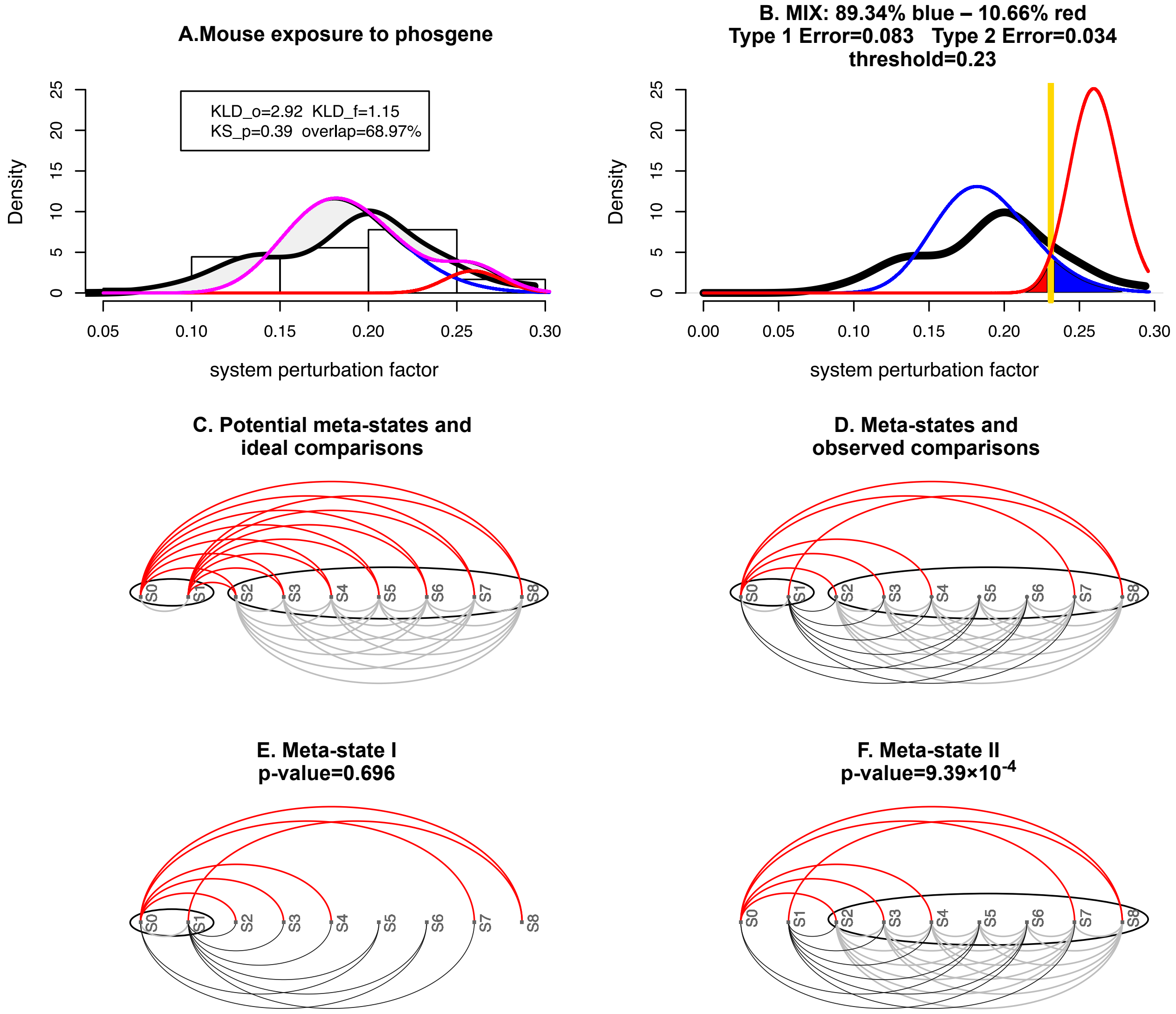

Figure 8: The results of QCD on real data of the mouse exposure to carbonyl chloride. Panel A shows the mixture (magenta line) of two gamma distributions (blue and red lines) that is fitted to the perturbation factors (histogram, and density - thick black line). The blue and red density lines are scaled using the mixture proportion. We evaluate the goodness of fit using three statistics: (i) the Kullback-Leibler divergence between the density of the observed perturbation and the density of the fitted mixture model (KLD_o-observed first, KLD_f-fitted first); (ii) the p-value of the Kolmogorov-Smirnov test (KS_p) between the observed perturbation and the fitted mixture model; and (iii) the overlap, which is the ratio between the intersection and union of the areas delimited by the observed (thick black) and fitted (magenta) density lines. Panel B shows the gamma mixture model used to separate small (blue line) and large perturbations (red line). The blue and red distributions which compose the mixture model are unscaled in this panel and the mixture proportion is reported. The yellow vertical line is the threshold used to separate the small and large perturbation factors. The null hypothesis is that there are no change intervals and therefore there are only small system perturbations (blue distribution). The Type 1 and Type 2 errors are marked by the blue and red areas, respectively. Panel C shows the potential meta-states (black ellipses) together with the ideal comparisons between the time points within these meta-states (red - large perturbation, gray - small perturbation). Panel D shows the same meta-states (black ellipses) together with the observed comparisons (red - large perturbation, gray and black - small perturbation). Black comparisons are between states from different meta-states (these are red in the ideal case). Panel E shows the comparisons considered for meta-state I. Panel F shows the comparisons considered for meta-state II.

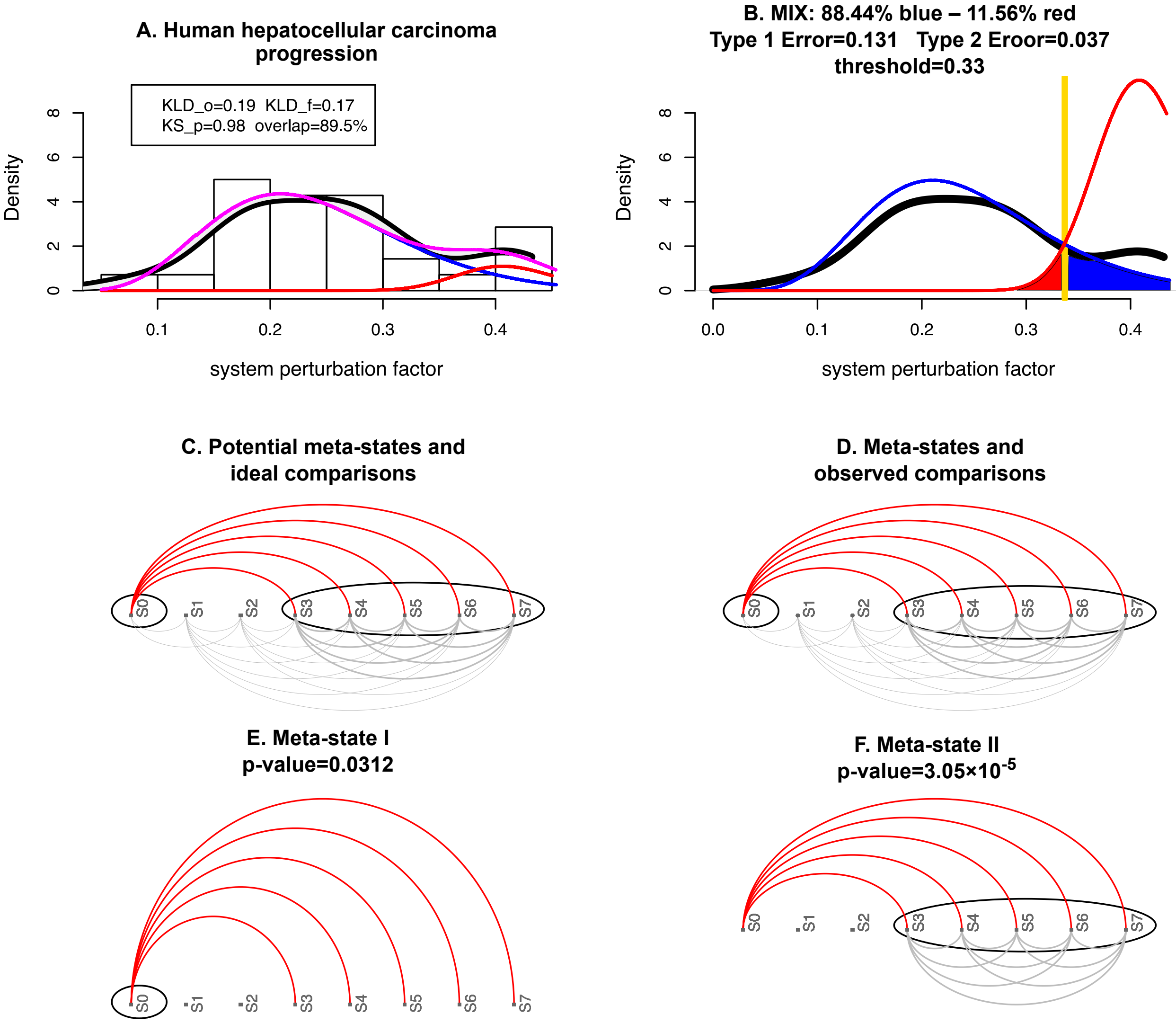

Figure 9: The results of QCD on real data for the human hepatitis C virus (HCV) to hepatocellular carcinoma (HCC) progression. Panel A shows the mixture (magenta line) of two gamma distributions (blue and red lines) that is fitted to the perturbation factors (histogram, and density - thick black line). The blue and red density lines are scaled using the mixture proportion. We evaluate the goodness of fit using three statistics: (i) the Kullback-Leibler divergence between the density of the observed perturbation and the density of the fitted mixture model (KLD_o-observed first, KLD_f-fitted first); (ii) the p-value of the Kolmogorov-Smirnov test (KS_p) between the observed perturbation and the fitted mixture model; and (iii) the overlap, which is the ratio between the intersection and union of the areas delimited by the observed (thick black) and fitted (magenta) density lines. Panel B shows the gamma mixture model used to separate small (blue line) and large perturbations (red line). The blue and red distributions which compose the mixture model are unscaled in this panel and the mixture proportion is reported. The yellow vertical line is the threshold used to separate the small and large perturbation factors. The null hypothesis is that there are no change intervals and therefore there are only small system perturbations (blue distribution). The Type 1 and Type 2 errors are marked by the blue and red areas, respectively. Panel C shows the potential meta-states (black ellipses) together with the ideal comparisons between the time points within these meta-states (red - large perturbation, gray - small perturbation). Panel D shows the same meta-states (black ellipses) together with the observed comparisons (red - large perturbation, gray and black - small perturbation). Black comparisons are between states from different meta-states (these are red in the ideal case). Panel E shows the comparisons considered for meta-state I. Panel F shows the comparisons considered for meta-state II.

2 Results of followup analysis for hepatitis C virus (HCV)-induced hepatocellular carcinoma (HCC) progression

To further investigate the results of our analysis in the case of HCC progression we identified the genes that change (absolute log2 fold change greater than 1) when comparing control to high-grade dysplasia and control to very advanced HCC. In the control versus high-grade dysplasia comparison there are 149 DE genes, while in the control versus very advanced HCC comparison there are 1,355 DE genes, which is almost an order of magnitude higher. This suggests that using the genes that are differentially expressed across the change interval, as opposed to the genes that are different between control and very advanced HCC, offers a more focused analysis. In essence, the comparison across the narrowest change interval targets the genes involved in the initial tumor formation, rather than all genes that change as a consequence of the cancer. The number of common DE genes among the two comparisons is 80, representing 53% of the initial 149 genes.

2.1 Followup analysis using the cancer gene census

We downloaded the curated list of cancer genes available in the cancer gene census [12] (accessible at: http://cancer.sanger.ac.uk/census). This list is is presented together with the catalogue of somatic mutations in cancer (COSMIC) [13] (accessible at: http://cancer.sanger.ac.uk/cosmic). We used this list of cancer genes to filter the 80 common genes. The result consists of two genes: CHEK2, a tumor suppressor, and FAT1, which is known to act both as a tumor suppressor as well as an oncogene. These are genes highly relevant to the condition under study considering CHEK2 mutations have been linked to various cancers [14, 15] and it has also been shown to be a mediator of a tumorigenic mechanism specifically in HCC [16]. In addition, FAT1 has been shown to have an oncogenic role in HCC [17, 18], as well as it has been identified as a biomarker in multiple cancers [19, 20]. Figure 10 shows the expression of CHEK2 and FAT1 over the disease progression stages. The expression of both genes increases with disease progression with a sharp increase taking place during the change interval, which may be a potential window for treatment.

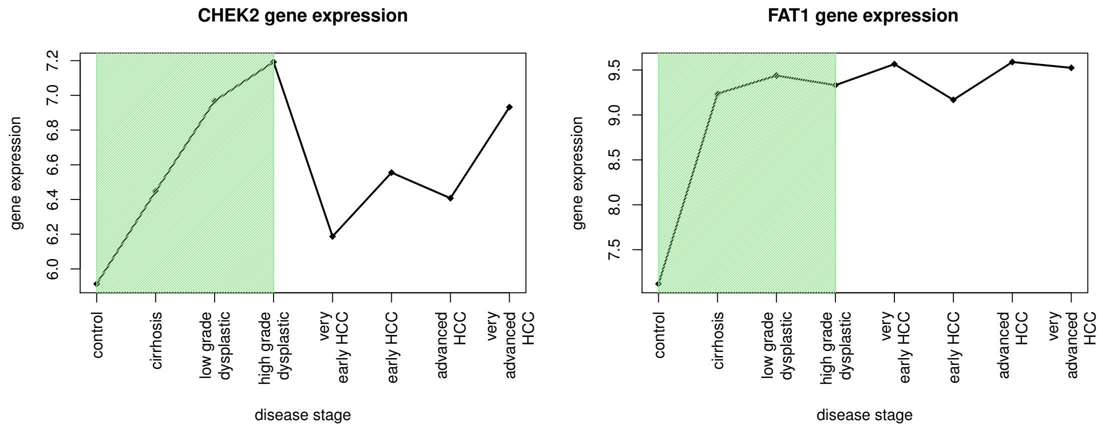

Figure 10: The log 2 expression of CHEK2 (left) and FAT1 (right) over 8 stages of disease progression from healthy to advanced hepatocellular carcinoma (HCC). Data from GEO (GSE6764) for the human hepatitis C virus (HCV) to HCC progression. The green shaded area is the change interval detected using this gene expression dataset and the viral carcinogenesis pathway from KEGG [8, 9, 10] (hsa05203). CHEK2 and FAT1 steadily increase throughout the disease stages with a sharp increase in expression during the change interval. CHEK2 and FAT1 could potentially be targeted for downregulation. CHEK2 is a tumor suppressor, and FAT1 is known to act both as a tumor suppressor as well as an oncogene. CHEK2 mutations have been linked to various cancers [14, 15]. FAT1 has been studied in HCC where it has been shown to have an oncogenic role [17, 18] and has been identified as a biomarker in multiple cancers [19, 20].

2.2 Followup analysis using KEGG pathways

The "Viral carcinogenesis pathway" from KEGG [8, 9, 10] was used to identify the change interval for the HCV induced HCC progression. To further investigate the results of QCD we used this pathway in a followup analysis. As mentioned above, we compared the control with the high-grade dysplasia stage and the control with the very advanced HCC stage and identified 80 common differentially expressed genes. We also used this pathway to filter the 80 common genes and obtained a "Viral carcinogenesis" gene set, which contains genes from the pathway that change at the onset of the disease. The result consists of two early growth response genes: EGR2 and EGR3. EGR2 has been shown to be an apoptosis promoter gene [21], which is downregulated by miRNAs in cancer [22, 23]. EGR3 has been shown to be involved in a number of cancers and the regulation of the immune response [24, 25, 26, 27] and has recently been linked to HCC where it was used to inhibit the growth of tumor cells [28]. Figure 11 shows the expression of EGR2 and EGR3 over the disease progression stages. The expression of both genes decreases with disease progression with the sharpest decrease taking place during the change interval.

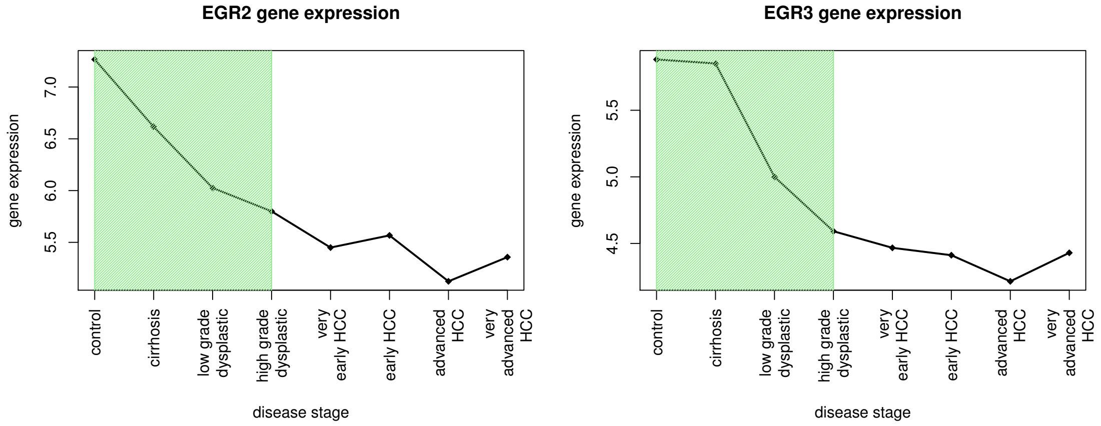

Figure 11: The log 2 expression of EGR2 (left) and EGR3 (right) over 8 stages of disease progression from healthy to advanced hepatocellular carcinoma (HCC). Data from GEO (GSE6764) for the human hepatitis C virus (HCV) to HCC progression. The green shaded area is the change interval detected using this gene expression dataset and the viral carcinogenesis pathway from KEGG [8, 9, 10] (hsa05203). EGR2 and EGR3 steadily decrease throughout the disease stages with a sharp decrease in expression during the change interval. EGR2 and EGR3 could potentially be targeted for over expression. EGR2 is known to apoptosis promoter gene [21] that is known to be downregulated by miRNAs in cancer [22, 23]. EGR3 is known to be involved in a number of cancers and the regulation of the immune response [24, 25, 26, 27] and has recently been linked to HCC where it was used to inhibit the growth of tumor cells [28].

3 QCD behavior under the null hypothesis

An important question for the proposed method is to demonstrate that the approach does not report qualitative changes in the case of experiments that do not involve any system perturbations (false positive changes).

The hypothesis is that if there is a change interval the system state comparisons will yield a mix of large and small system perturbations. Small system perturbation are expected when comparing system states before the change interval or system states after the change interval. Large system perturbations are expected when comparing system states before the change interval with states after the change interval. Therefore we used a mixture of two gamma distributions, one for the comparisons in which the system is unperturbed (which is also the null hypothesis) and another for comparisons in which the system is perturbed. The mixture model will be initialized with two distributions having the mode the minimum and maximum of the perturbation factors. The mixture model fitting will provide two distributions that best fit the data

together with a percentage which estimates how much of the observed data comes from each of these two distributions. If any of the distributions has a percentage of less than 10%, we consider that there is only one distribution and therefore we will not report any significant change.

In order to investigate the behavior of this approach under the null distribution, when the system is only affected by random noise and small random fluctuations, we used the time-course data from the control samples involved in the perturbation experiments above.

3.1 Control data from the fruit fly ethanol exposure experiment

The study by Kong et al. [29] on fruit fly exposure to ethanol contains both condition and control time course data. The experiment spans 3.5 hours (210 min) of recovery after a 30 min ethanol exposure sedating up to 75% of the flies and is sampled at 8 time points. The time-points include one control before exposure, one at 0h right after exposure, and every 30 minutes after that up to 3.5h with a missing data point at 2.5h (150 min) which was not provided in the dataset. Treatment conditions used in this experiment were exposure to humidified air or ethanol vapor (60%) for 30 min, and then recovery for up to 210 minutes [29]. Samples exposed to humidified air are the control samples. Figure 12 presents the first two steps of the QCD method on the control data. A mixture of two gamma distributions is fitted to the perturbation factors computed for all time points comparisons on the control data. The large perturbations (red distribution) contribute only 2.73% of the mixture. In other words, the comparisons between system states show mostly small perturbation, which means there is no significant system change.

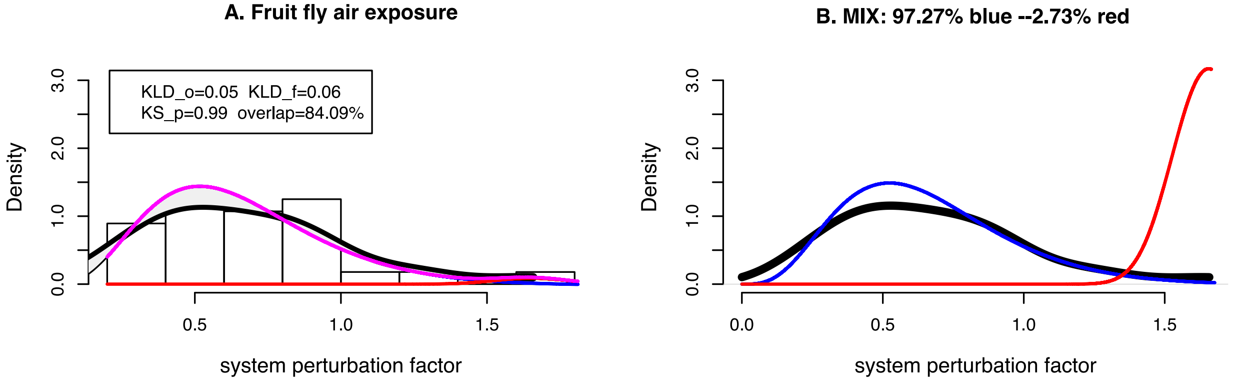

Figure 12: The results of QCD on real data of the fruit fly exposure to air. Panel A shows the mixture (magenta line) of two gamma distributions (blue and red lines) that is fitted to the observed perturbation factors (histogram, and density - thick black line). The blue and red density lines are scaled using the mixture proportions. We evaluate the goodness of fit using three statistics: (i) the Kullback-Leibler divergence, which is computed between the density of the observed perturbation and the density of the fitted mixture model (KLD_o-observed first, KLD_f-fitted first); (ii) the p-value of the Kolmogorov-Smirnov test (KS_p) between the observed perturbation and a sample of the fitted mixture model; and (iii) the overlap, which is the ratio between the intersection and union of the areas delimited by the observed (thick black) and fitted (magenta) density lines. The blue and red distributions which compose the mixture model are unscaled in this panel and the mixture proportion is reported. The red distribution (large perturbation) contributes only 2.73% of the mixture. In other words, the comparisons between system states show mostly small perturbation, which means there is no significant system change.

3.2 Control data from the mouse phosgene exposure experiment

In the study by Sciuto et al. [7], mice were exposed to 32 mg of phosgene per cubic meter for 20 min and samples were collected from lung tissue at 9 time points: untreated (0), 30 min, 1, 4, 8, 12, 24, 48, 72 hours after exposure. As a control, samples were collected at the same time points from mice exposed to air. Figure 13 presents the first two steps of the QCD method on the control data. A mixture of two gamma distributions is fitted to the perturbation factors computed for all time points comparisons on the control data. Results show that the distribution of large system perturbations contributes less than 10% of the mixture. In other words, the comparisons between system states show mostly small perturbation, which means there is no significant system change.

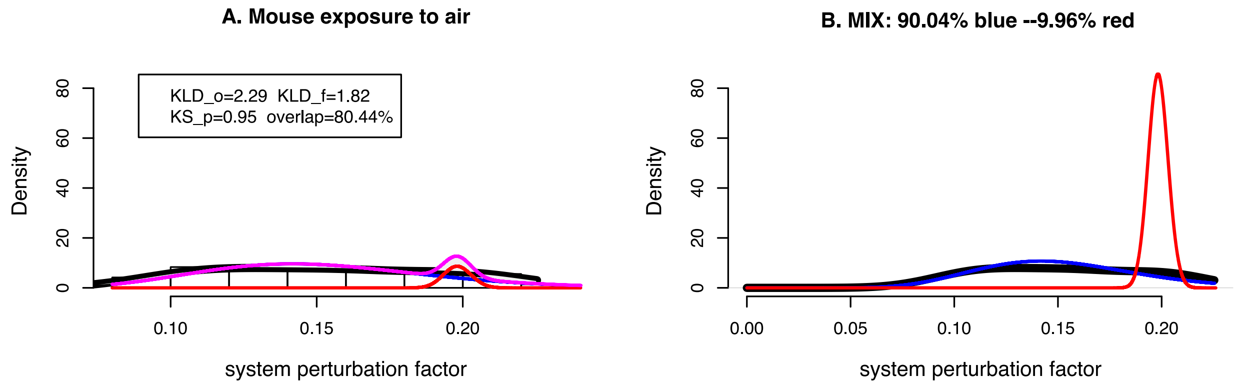

Figure 13: The results of QCD on real data of the mouse exposure to air. Panel A shows the mixture (magenta line) of two gamma distributions (blue and red lines) that is fitted to the perturbation factors (histogram, and density - thick black line). The blue and red density lines are scaled using the mixture proportions. We evaluate the goodness of fit using three statistics: (i) the Kullback-Leibler divergence, which is computed between the density of the observed perturbation and the density of the fitted mixture model (KLD_o-observed first, KLD_f-fitted first); (ii) the p-value of the Kolmogorov-Smirnov test (KS_p) between the observed perturbation and a sample of the fitted mixture model; and (iii) the overlap, which is the ratio between the intersection and union of the areas delimited by the observed (thick black) and fitted (magenta) density lines. The blue and red distributions which compose the mixture model are unscaled in this panel and the mixture proportion is reported. The red distribution (large perturbation) makes up for only 9.96% of the mixture. In other words, the comparisons between system states show mostly small perturbation, which means there is no significant system change.

3.3 Behavior on random data

An important requirement is to demonstrate that the approach does not report significant changes in random data. In order to investigate this, we generated 10,000 perturbation factors using random samples from the *E coli* flagellum building data. We select randomly 10 time points out of the 21 available, and we compute a perturbation factor comparing the average of the selected 10 time points with the average of the other 11 time points. This process generates 10,000 random perturbation factors. We fit a mixture of two gamma distributions to these data (see Fig. 14). The large perturbations (red distribution) make up for only 2.64% of the mixture. In other words, the comparisons between system states show mostly small perturbation, which means there is no significant system change. Thus, QCD does not report any false positives when the data is random.

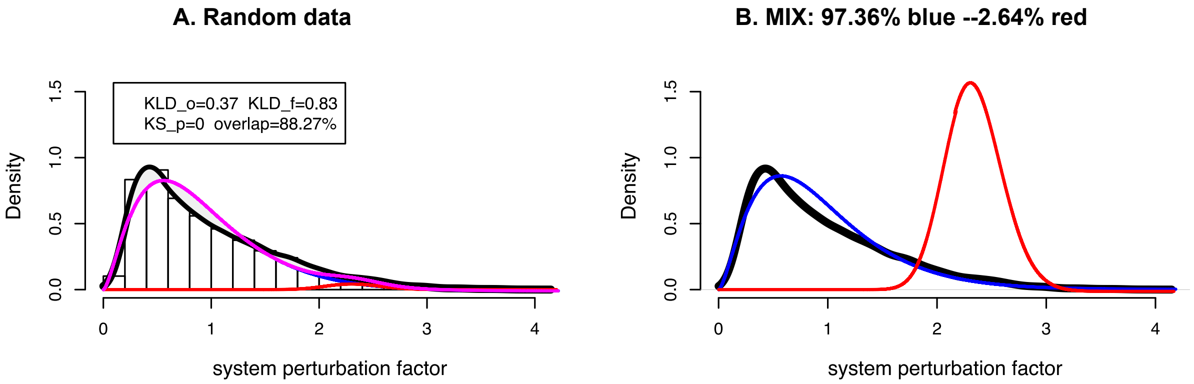

Figure 14: The results of QCD on random data generated using the *E. coli* flagellum building synthetic data. Randomly selected groups of 10 (out of a total of 21) system states are compared to the group of remaining 11 system states to generate random perturbation values. Data is generated using 10,000 iterations. Panel A shows the mixture (magenta line) of two gamma distributions (blue and red lines) that is fitted to the perturbation factors (histogram, and density - thick black line). The blue and red density lines are scaled using the mixture proportion. We evaluate the goodness of fit using three statistics: (i) the Kullback-Leibler divergence, which is computed between the density of the observed perturbation and the density of the fitted mixture model (KLD_o-observed first, KLD_f-fitted first); (ii) the p-value of the Kolmogorov-Smirnov test (KS_p) between the observed perturbation and a sample of the fitted mixture model; and (iii) the overlap, which is the ratio between the intersection and union of the areas delimited by the observed (thick black) and fitted (magenta) density lines. Panel B shows how the gamma mixture model is used to separate small (blue line) and large perturbations (red line). The blue and red distributions which compose the mixture model are unscaled in this panel and the mixture proportion is reported. The red distribution (large perturbation) makes up for only 2.64% of the mixture. In other words, the comparisons between system states show mostly small perturbation, which means there is no significant system change.

4 Comparison: unimodal vs bimodal fit of gamma distributions

When we applied the fit of one and two gamma distributions to all 8 case studies, there are five cases in which two gamma distributions better fit the data and three cases in which a single gamma distribution gives the better fit: yeast, fruit fly and mouse (see Table 1). Notably, all the synthetic data (which models the phenomenon without noise) as well as the data for the human hepatocellular carcinoma progression and fruit fly pupariation were better fit by a mixture of two gamma distributions. In the case of the same comparison under the null hypothesis, when there is no true change, two out of the three cases show a better fit of a model using two gamma distributions (see Table 2). For the case of mouse exposure to air the results show a tie, two metrics showing a better fit for the model with two gamma distributions and the other two metrics showing a better fit for a the model with a single gamma distribution. Importantly, the use of the models with two gamma distributions did not lead to false findings. Even though we fit a model with two gamma distributions, the approach correctly concluded that there were no qualitative changes in those data sets. In summary, using the model with two distributions yields results that are overall better across 8 data sets and 3 null hypotheses experiments and therefore, we retained this model.

The R mixtool package and the gammamixEM function were used to fit one or two gamma distributions to the data (system perturbation factors for all state comparisons).

**Results of the fit of 1 and 2 Gamma distribution - gammamixEM**						
**Organism**	**Phenomena**	**Overlap (%)**	**KS**	**KLD_o**	**KLD_f**	**No. distr.**
E. coli	Flagella building	72.53	0.42	0.01	0.01	2
		62.35	$8.5 * 10^{-5}$	0.01	0.01	1
B. subtilis	Sporulation	81.54	0.81	0.02	0.03	2
		63.43	$9.46 * 10^{-6}$	0.03	0.03	1
C. elegans	Avoidance reflex	74.74	0.98	0.03	0.04	2
		NA	NA	NA	NA	1
S. cerevisiae	Sporulation	73.37	0.99	0.08	0.08	2
		81.55	0.99	0.04	0.05	1
D. melanogaster	Pupariation	75.7	0.86	0.07	0.07	2
		73.78	0.06	0.07	0.08	1
	Acute ethanol	80.82	0.94	0.04	0.06	2
	exposure	86.87	0.95	0.04	0.06	1
M. musculus	Carbonyl chloride	68.97	0.39	2.92	1.15	2
	exposure	81.33	0.73	1.17	0.57	1
H. sapiens	HCV induced	89.5	0.98	0.17	0.19	2
	HCC progression	87.24	0.88	0.24	0.19	1

Table 1: Summary of the results for the Gamma distribution fit for two or one gamma distributions for each of the 8 phenomena. Each row is one phenomenon, and for each row we have two sets of information (sub-rows) with the results for the fit of two or one gamma distributions. The metrics on columns 3-6 are: % overlap of the observed and fitted distributions, p-value of the Kolmogorov-Smirnov test between the observed values and a sample of the fitted distribution, Kullback-Leibler divergence (observed first or fitted first) between the observed and fitted distributions. In the case of the *C. elegans* avoidance reflex, we used for the result of fitting one distribution because the gammamixEM returned an error and did not fit any distribution to the data. In five case studies two gamma distributions show a better fit of the data. In three case studies a single gamma distribution gives the better fit. Notably, all the synthetic data (which models the phenomenon without noise) as well as the data for the human hepatocellular carcinoma progression and fruit fly pupariation were better fit by a mixture of two gamma distributions. The results of this comparison support the use of a mixture of two gamma distributions to model the data in order to identify large and small perturbations.

Results of the fit of 1 and 2 Gamma distribution - gammamixEM						
**Organism**	**Phenomena**	**Overlap (%)**	**KS**	**KLD_o**	**KLD_f**	**No. distr.**
E. coli	Flagella building - random data	88.27	0	0.37	0.83	2
		87.89	$6.66 * 10^{-16}$	0.38	0.93	1
D. melanogaster	Air exposure	84.9	0.99	0.05	0.06	2
		84.05	0.94	0.06	0.08	1
M. musculus	Air exposure	80.44	0.95	2.29	1.82	2
	exposure	77.06	0.89	2.01	0.88	1

Table 2: Summary of the results for the gamma distribution fit for two or one gamma distributions under the null distribution. Each row is one phenomenon, and for each row we have two sets of information (sub-rows) with the results for the fit of two or one gamma distributions. The metrics on columns 3-6 are: % overlap of the observed and fitted distributions, p-value of the Kolmogorov-Smirnov test between the observed values and a sample of the fitted distribution, Kullback-Leibler divergence (observed first or fitted first) between the observed and fitted distributions. In two out of three case studies two gamma distributions show a better fit the data. For the case of the mouse exposure to air the results show a tie, two metrics showing a better fit of two gamma distributions and the other two metrics showing a better fit for a single gamma distribution. The results of this comparison support the use of a mixture of two gamma distributions to model the data in order to identify large and small perturbations.

References

[1] Drăghici, S., Khatri, P., Tarca, A.L., Amin, K., Done, A., Voichiţa, C., Georgescu, C., Romero, R.: A systems biology approach for pathway level analysis. Genome Research **17**(10), 1537–1545 (2007)

[2] Tarca, A.L., Drăghici, S., Khatri, P., Hassan, S.S., Mittal, P., Kim, J.-S., Kim, C.J., Kusanovic, J.P., Romero, R.: A novel signaling pathway impact analysis (SPIA). Bioinformatics **25**(1), 75–82 (2009)

[3] Voichiţa, C., Drăghici, S.: ROntoTools: R Onto-Tools Suite. (2013). R package

[4] Voichiţa, C., Donato, M., Drăghici, S.: Incorporating gene significance in the impact analysis of signaling pathways. In: Machine Learning and Applications (ICMLA), 2012 11th International Conference On, vol. 1, pp. 126–131. IEEE, Boca Raton, FL, USA (2012)

[5] Sciuto, A.M., Hurt, H.H.: Therapeutic treatments of phosgene-induced lung injury. Inhalation Toxicology **16**(8), 565–580 (2004)

[6] Sciuto, A.M., Lee, R.B., Forster, J.S., Cascio, M.B., Clapp, D.L., Moran, T.S.: Temporal changes in respiratory dynamics in mice exposed to phosgene. Inhalation Toxicology **14**(5), 487–501 (2002)

[7] Sciuto, A.M., Phillips, C.S., Orzolek, L.D., Hege, A.I., Moran, T.S., Dillman, J.F.: Genomic analysis of murine pulmonary tissue following carbonyl chloride inhalation. Chemical Research in Toxicology **18**(11), 1654–1660 (2005)

[8] Kanehisa, M., Goto, S.: KEGG: Kyoto encyclopedia of genes and genomes. Nucleic Acids Research **28**(1), 27–30 (2000)

[9] Kanehisa, M., Sato, Y., Furumichi, M., Morishima, K., Tanabe, M.: New approach for understanding genome variations in KEGG. Nucleic Acids Research **47**(D1), 590–595 (2018)

[10] Kanehisa, M.: Toward understanding the origin and evolution of cellular organisms. Protein Science **28**(11), 1947–1951 (2019)

[11] Liu, R., Li, M., Liu, Z.-P., Wu, J., Chen, L., Aihara, K.: Identifying critical transitions and their leading biomolecular networks in complex diseases. Scientific Reports **2** (2012). Article number: 813

[12] Futreal, P.A., Coin, L., Marshall, M., Down, T., Hubbard, T., Wooster, R., Rahman, N., Stratton, M.R.: A census of human cancer genes. Nature Reviews Cancer **4**(3), 177–183 (2004)

[13] Forbes, S., Beare, D., Bindal, N., Bamford, S., Ward, S., Cole, C., Jia, M., Kok, C., Boutselakis, H., De, T., Sondka, Z., Ponting, L., Stefancsik, R., Harsha, B., Tate, J., Dawson, E., Thompson, S., Jubb, H., Campbell, P.: COSMIC: high-resolution cancer genetics using the catalogue of somatic mutations in cancer. Current Protocols in Human Genetics, 10–11 (2016)

[14] Vahteristo, P., Bartkova, J., Eerola, H., Syrjäkoski, K., Ojala, S., Kilpivaara, O., Tamminen, A., Kononen, J., Aittomäki, K., Heikkilä, P., Holli, K., Blomqvist, C., Bartek, J., Kallioniemi, O.-P., Nevanlinna, H.: A CHEK2 genetic variant contributing to a substantial fraction of familial breast cancer. The American Journal of Human Genetics **71**(2), 432–438 (2002)

[15] Dong, X., Wang, L., Taniguchi, K., Wang, X., Cunningham, J.M., McDonnell, S.K., Qian, C., Marks, A.F., Slager, S.L., Peterson, B.J., Smith, D.I., Cheville, J.C., Blute, M.L., Jacobsen, S.J., Schaid, D.J., Tindall, D.J., Thibodeau, S.N., Liu, W.: Mutations in CHEK2 associated with prostate cancer risk. The American Journal of Human Genetics **72**(2), 270–280 (2003)

[16] Neumann, O., Kesselmeier, M., Geffers, R., Pellegrino, R., Radlwimmer, B., Hoffmann, K., Ehemann, V., Schemmer, P., Schirmacher, P., Lorenzo Bermejo, J., Longerich, T.: Methylome analysis and integrative profiling of human HCCs identify novel protumorigenic factors. Hepatology **56**(5), 1817–1827 (2012)

[17] Pereira, B., Chin, S.-F., Rueda, O.M., Vollan, H.-K.M., Provenzano, E., Bardwell, H.A., Pugh, M., Jones, L., Russell, R., Sammut, S.-J., *et al.*: The somatic mutation profiles of 2,433 breast cancers refines their genomic and transcriptomic landscapes. Nature Communications **7**, 11479 (2016)

[18] Valletta, D., Czech, B., Spruss, T., Ikenberg, K., Wild, P., Hartmann, A., Weiss, T.S., Oefner, P.J., Müller, M., Bosserhoff, A.-K., Hellerbrand, C.: Regulation and function of the atypical cadherin FAT1 in hepatocellular carcinoma. Carcinogenesis **35**(6), 1407–1415 (2014)

[19] De Bock, C., Ardjmand, A., Molloy, T., Bone, S., Johnstone, D., Campbell, D., Shipman, K., Yeadon, T., Holst, J., Spanevello, M., Nelmes, G., Catchpoole, R., Lincz, L., Boyd, A., Burns, G., Thorne, R.: The Fat1 cadherin is overexpressed and an independent prognostic factor for survival in paired diagnosis–relapse samples of precursor B-cell acute lymphoblastic leukemia. Leukemia **26**(5), 918–926 (2012)

[20] Wang, L., Lyu, S., Wang, S., Shen, H., Niu, F., Liu, X., Liu, J., Niu, Y.: Loss of FAT1 during the progression from DCIS to IDC and predict poor clinical outcome in breast cancer. Experimental and Molecular Pathology **100**(1), 177–183 (2016)

[21] Unoki, M., Nakamura, Y.: EGR2 induces apoptosis in various cancer cell lines by direct transactivation of BNIP3L and BAK. Oncogene **22**(14), 2172–2185 (2003)

[22] Wu, Q., Jin, H., Yang, Z., Luo, G., Lu, Y., Li, K., Ren, G., Su, T., Pan, Y., Feng, B., Xue, Z., Wang, X., Fan, D.: miR-150 promotes gastric cancer proliferation by negatively regulating the pro-apoptotic gene EGR2. Biochemical and biophysical research communications **392**(3), 340–345 (2010)

[23] Liu, X., Shi, H., Liu, B., Li, J., Liu, Y., Yu, B.: miR-330-3p controls cell proliferation by targeting early growth response 2 in non-small-cell lung cancer. Acta biochimica et biophysica Sinica **47**(6), 431–440 (2015)

[24] Inoue, A., Omoto, Y., Yamaguchi, Y., Kiyama, R., Hayashi, S.I.: Transcription factor EGR3 is involved in the estrogen-signaling pathway in breast cancer cells. Journal of Molecular Endocrinology **32**(3), 649–661 (2004)

[25] Safford, M., Collins, S., Lutz, M.A., Allen, A., Huang, C.-T., Kowalski, J., Blackford, A., Horton, M.R., Drake, C., Schwartz, R.H., Powell, J.D.: Egr-2 and Egr-3 are negative regulators of T cell activation. Nature Immunology **6**(5), 472–480 (2005)

[26] Pio, R., Jia, Z., Baron, V.T., Mercola, D.: Early growth response 3 (Egr3) is highly over-expressed in non-relapsing prostate cancer but not in relapsing prostate cancer. PLoS One **8**(1), 54096 (2013)

[27] Cheng, H., Hao, S., Liu, Y., Pang, Y., Ma, S., Dong, F., Xu, J., Zheng, G., Li, S., Yuan, W., Cheng, T.: Leukemic marrow infiltration reveals a novel role for Egr3 as a potent inhibitor of normal hematopoietic stem cell proliferation. Blood **126**(11), 1302–1313 (2015)

[28] Zhang, S., Xia, C., Xu, C., Liu, J., Zhu, H., Yang, Y., Xu, F., Zhao, J., Chang, Y., Zhao, Q.: Early growth response 3 inhibits growth of hepatocellular carcinoma cells via upregulation of fas ligand. International Journal of Oncology **50**(3), 805–814 (2017)

[29] Kong, E.C., Allouche, L., Chapot, P.A., Vranizan, K., Moore, M.S., Heberlein, U., Wolf, F.W.: Ethanol-regulated genes that contribute to ethanol sensitivity and rapid tolerance in Drosophila. Alcoholism: Clinical and Experimental Research **34**(2), 302–316 (2009)
